# Bandgap-Engineered High-Efficiency Blue- and Green-Emitting CdZnSeS/ZnS Quaternary Alloyed Core/Shell Colloidal Nanoplatelets for High-Performance Light-Emitting Devices

**DOI:** 10.1021/acsami.5c04630

**Published:** 2025-06-02

**Authors:** Aisan Khaligh, Savas Delikanli, Betul Canimkurbey, Farzan Shabani, Furkan Isik, Hilmi Volkan Demir

**Affiliations:** † UNAM-Institute of Materials Science and Nanotechnology and the National Nanotechnology Research Center, Department of Electrical and Electronics Engineering, Department of Physics, Bilkent University, Ankara 06800, Turkey; ‡ LUMINOUS! Centre of Excellence for Semiconductor Lighting and Displays, The Photonics Institute, School of Electrical and Electronic Engineering, School of Physical and Mathematical Sciences, School of Materials Science and Engineering, Nanyang Technological University, Singapore 639798, Singapore; § Department of Physics, Polatlı Faculty of Science and Letters, Ankara Hacı Bayram Veli University, Ankara 06900, Turkey

**Keywords:** colloidal nanoplatelets, heterostructures, cation exchange, blue emission, light-emitting diodes

## Abstract

Developing solution-processed blue emitters with high stability and photoluminescence quantum yield (PL-QY) is strongly desired for advanced optoelectronic devices. However, achieving high-efficiency blue emitters has been challenging, as the growth of shell layers required for passivation of nonradiative recombination pathways induces a considerable red shift toward longer wavelengths in colloidal nanocrystals. To address this limitation, in this work, we propose and demonstrate a meticulous synthetic approach to develop highly efficient CdZnSeS/ZnS quaternary alloyed core/shell nanoplatelets (NPLs) with controllable shell thickness and core composition, exhibiting blue or green emission, depending on the core composition. Starting with the CdSe_0.7_S_0.3_ alloyed core NPLs, a thin ZnS shell was first grown through the hot injection (HI) technique, followed by a Cd-to-Zn cation-exchange (CE) reaction, which blue-shifts the absorption/emission peaks. Then, a wide-gap ZnS shell was grown a second time to passivate the surface and obtain high-efficiency thick NPLs with a PL-QY of >70% over a broad spectrum (ca. 460–560 nm). Despite the increased thickness, the thick-shell quaternary NPLs exhibit a minimal PL red shift. The blue light-emitting diode (LED) device fabricated using these bandgap-engineered NPLs demonstrates an exceptionally high external quantum efficiency (EQE) of 11.3% at 482 nm with a low turn-on voltage (*V*_T_) of less than 2.5 V, and a maximum luminance (*L*_max_) of 12,451 cd/m^2^. These advanced heterostructures of NPLs with highly efficient tunable emission in blue and green provide a great platform for developing high-performance light-emitting devices, especially for LEDs and lasers.

## Graphical abstract

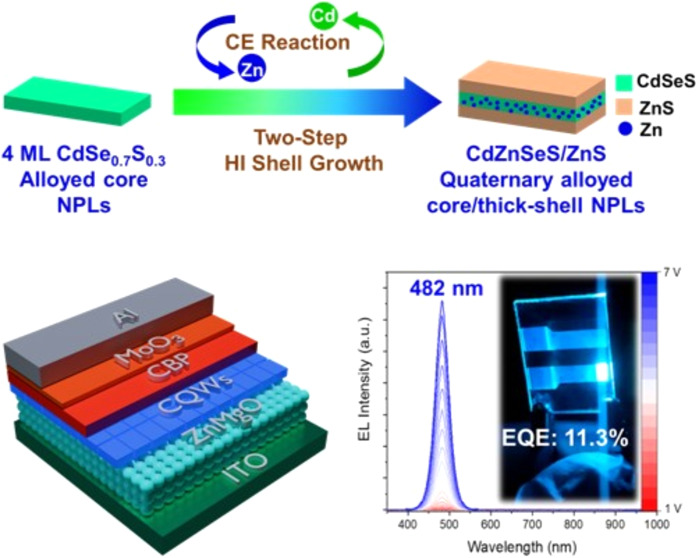

## Introduction

Semiconductor colloidal quantum wells (CQWs), more commonly referred to as nanoplatelets (NPLs), represent a unique class of two-dimensional nanomaterials with their tunable atomic-level thickness and large lateral dimensions.^1–6^ This unique structure allows for remarkable optical and electronic properties,^7–13^ making semiconductor NPLs a great platform for high-performance light-emitting and -absorbing applications, including those of light-emitting diodes (LEDs),^6,14–18^ lasers,^5,19–22^ solar light concentrators,^23^ photodetectors,^24,25^ and photocatalysts.^26^

CdSe NPLs are among the most extensively researched NPLs for the design and synthesis of advanced heterostructures.^13,16,17,27–30^ Since the quantum confinement in NPLs only occurs along the vertical axis, they exhibit discrete emission and absorption profiles, and this makes it challenging to achieve a continuous range of colors, especially at shorter wavelengths.^2,31–34^ So far, several methods have been reported to fine-tune the emission of CdSe NPLs, enhance their emission stability, and increase their photoluminescence quantum yield (PL-QY), including shell growth,^13,30^ crown growth,^17,18,35,36^ core/crown/shell heterostructures,^37^ as well as alloying^18,38^ and doping.^33,39,40^

Alloying of NPLs offers a logical pathway to tune the excitonic properties of CdSe NPLs, particularly toward the blue spectral region. Examples in the literature have mostly focused on the homogeneously alloyed CdSe_*x*_S_1–*x*_ core NPLs, demonstrating relatively tunable emission spectra from blue to green by adjusting the sulfur composition.^18,35,38,41^ However, these CdSe_*x*_S_1–*x*_ core-only NPLs exhibit low PL-QY of merely 10–20% and limited emission tunability in the range of ca. 480–510 nm, which largely hinders their electroluminescence applications.^38^ Recently, our research group has developed blue-emitting four-monolayer (ML) CdSe_*x*_S_1–*x*_/CdS alloyed core/crown NPLs emitting in the range of ∼462 to 487 nm with enhanced PL-QY up to ∼60%. The blue-emitting NPL-LEDs fabricated with these core/crown heterostructures showed external quantum efficiency (EQE) of ∼0.06% and a maximum luminance (*L*_max_) of only 12 cd/m^2^.^18^ In another study, Hu et al. demonstrated blue-emitting 3.5 ML CdSeS/CdS core/crown NPL-LEDs exhibiting an EQE of 1.6% and an *L*_max_ of 46 cd/m^2^.^42^

More recently, cation exchange (CE) between Cd and Zn has proven to be a promising strategy for designing blue-emitting heterostructures of colloidal NPLs, which are difficult to synthesize due to the low reactivity of Zn precursors.^43^ Earlier, Yoon et al. synthesized CdZnSe/ZnS core/shell NPLs through a Cd-to-Zn CE reaction on the CdSe/ZnS core/shell NPLs. The resulting NPLs exhibited a PL-QY of less than 60% in the blue region.^15^ Additionally, they reported a cyan-emitting LED with an *L*_max_ of 11,400 cd/m^2^, without providing the efficiency, EQE.^15^

Despite these great advances, the device performance of blue-emitting colloidal NPL-LEDs still lags significantly behind that of their red and green counterparts. One of the major challenges in the development of blue-emitting and even green-emitting CdSe-based core/shell heterostructures is the precise control over the size and thickness of the NPLs, as even small variations in thickness can significantly affect the electronic structure, leading to considerable shifts in their emission wavelength. Additionally, growing a thin shell often fails to provide sufficient surface passivation, leaving the core–shell NPLs vulnerable to environmental degradation and the formation of trap states. Consequently, developing efficient blue-emitting CdSe-based NPLs with long-term photostability and sufficient surface passivation remains a critical challenge.

In this study, we present the synthesis, optical characterization, and structural characterization of highly efficient blue- and green-emitting CdZnSeS/ZnS quaternary alloyed core/thick-shell NPLs with PL-QY of >70% to be readily utilized in optoelectronic devices. The PL-QYs of these green- and blue-emitting core/shell NPLs are among the highest reported for colloidal NPLs to date. CdZnSeS/ZnS alloyed core/shell NPLs were obtained through a meticulous four-step synthetic pathway by first synthesizing CdSe_0.7_S_0.3_ NPLs as seeds, then growing a thin ZnS shell on the seed NPLs before the Cd-to-Zn CE reaction, and finally growing a second ZnS thick shell to sufficiently passivate the surface and enhance the efficiency. This approach allows us to adjust the emission wavelength (particularly toward the blue spectral side) by controlling the core composition as well as the thickness of the resulting NPLs through the two-step HI shell coating, while preserving their inherent shape and crystal structure as well as maintaining the color of cation-exchanged NPLs’ emission even after the shell thickening. Finally, we fabricated a highly efficient blue-emitting NPL-LED device using CdZnSeS/ZnS quaternary alloyed core/thick-shell NPLs, which surpasses other CdSe-based blue NPL-LEDs reported in the literature^15,18,38,42,44^ and is comparable to the most recently reported blue NPL-LEDs.^45^

## Results and Discussion

Driven by the aim of developing efficient and high-performance blue-emitting CdSe-based core/shell NPLs with enhanced stability and optical properties, we designed and synthesized CdZnSeS/ZnS quaternary alloyed core/shell heterostructures through a multistep synthesis procedure. The schematic of the four-step synthetic approach to obtain the final NPLs and the corresponding absorption and photoluminescence spectra of the synthesized NPLs are presented in Figure 1a–e. The synthetic route illustrated in Figure 1a involves the synthesis of 4 ML of CdSe_0.7_S_0.3_ alloyed core NPLs as the seed followed by growing a ZnS HI thin shell. Then, a Cd-to-Zn CE reaction was conducted in the core of the NPLs, and finally, the thickness of the quaternary alloyed CdZnSeS/ZnS core/thin-shell NPLs was increased by a secondary HI shell coating to achieve a successful passivation. The detailed synthesis procedures of all of the NPLs are provided in the Materials and Methods section.

**Figure 1:**
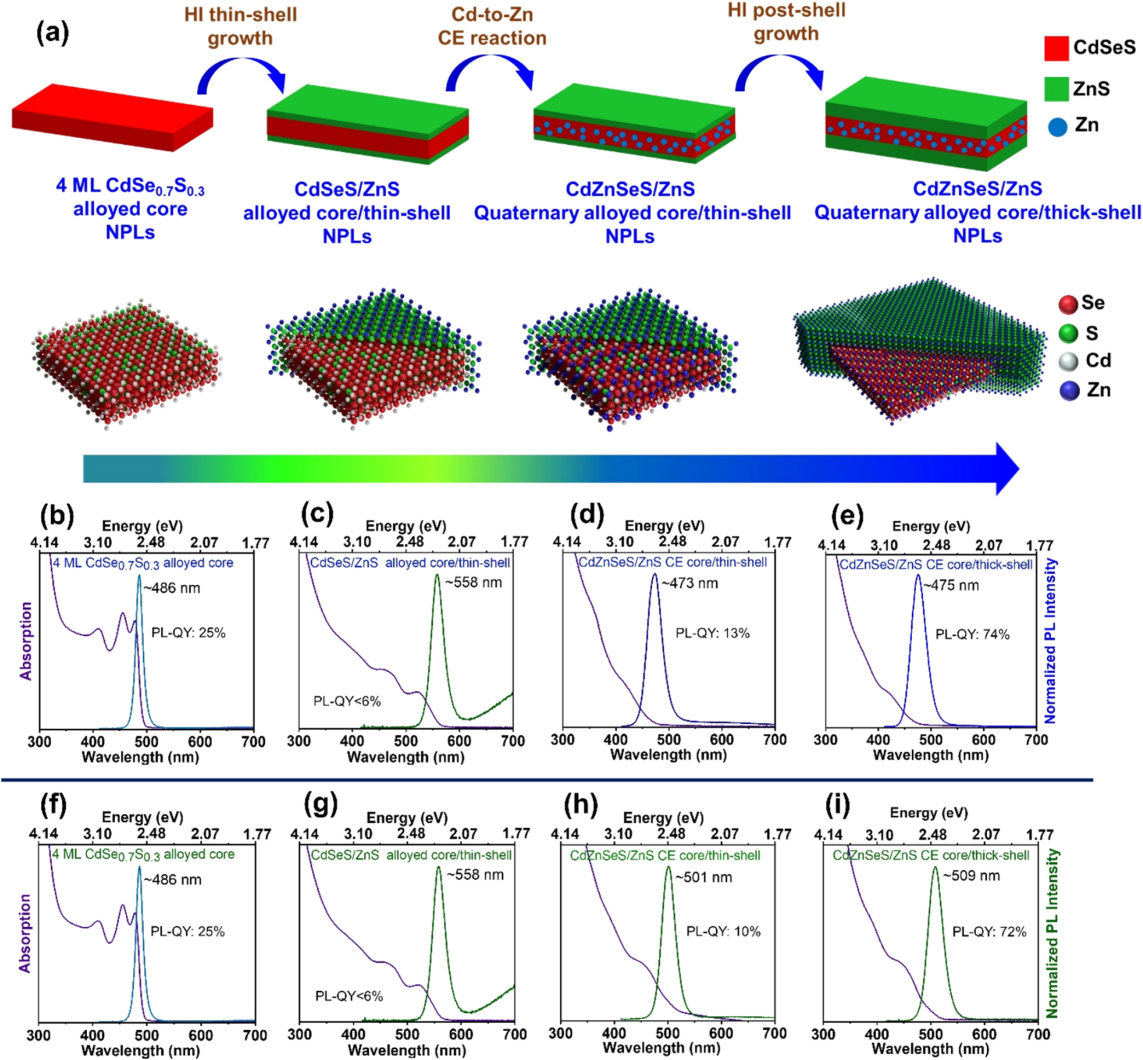
(a) Schematic representation of the four-step synthesis pathway toward the final CdZnSeS/ZnS quaternary alloy core/thick-shell NPLs. First, the CdSe_0.7_S_0.3_ core was passivated with a ZnS thin shell through the HI shell growth method. Then, Cd-to-Zn CE reaction was conducted in the core domain of the core/thin-shell NPLs followed by growing the final HI shell coating. Absorption and photoluminescence spectra of the synthesized NPLs through the four-step synthesis pathway: (b, f) alloyed core, (c, g) alloyed core/thin-shell, (d, h) quaternary alloyed core/thin-shell after CE, and (e, i) final quaternary alloyed core/thick-shell NPLs for the blue-emitting NPLs (first row) and green-emitting NPLs (second row).

We first started with the synthesis of the core NPLs as the base material, which were designed to emit in the blue spectral side to ensure that the emission peak of the core/thin-shell NPLs would remain in the green region, before the CE. While 3 ML CdSe NPLs could serve as core material owing to their blue emission, poor control over their large lateral size results in low colloidal stability and their tendency to roll causes strain and hence trap sites, which adversely affects their performance in optoelectronic devices. Additionally, the CE reaction on such thin NPLs causes the loss of their 2D shape.^46–48^ To address these limitations, alloying of the green-emitting 4 ML CdSe NPLs with sulfur is considered to shift the emission spectra toward the blue spectral side.^18^ Here, we synthesized 4 ML alloyed CdSe_0.7_S_0.3_ NPLs with blue-shifted emission and absorption spectra compared to those of the CdSe NPLs (Figure S1, SI) and used them as the starting material for the subsequent HI shell growth and CE processes. 4 ML CdSe_0.7_S_0.3_ alloyed core NPLs exhibit two absorption features at 477 and 455 nm associated with the electron-heavy hole and electron-light hole excitonic transitions, respectively, with the emission peak centered at 486 nm, yielding a Stokes shift of 9 nm (Figure 1b). These 4 ML CdSe_0.7_S_0.3_ alloyed core NPLs exhibit a full width at half-maximum (fwhm) of 15 nm and a PL-QY of 25%.

With the core NPLs in hand, we set out to conduct the Cd-to-Zn direct CE reaction. It has been reported that the core-only NPLs are unable to retain their 2D structure throughout the CE reaction because of the thinness of core NPLs compared to the reaction zone.^49^ Hence, a ZnS shell was first grown on the CdSe_0.7_S_0.3_ alloyed core NPLs at this stage through the HI shell coating technique to facilitate the Cd-to-Zn CE reaction and provide sufficient thermal and chemical stability for a high-temperature CE reaction, as the CE process did not occur in the CdSe_0.7_S_0.3_ core-only NPLs. However, the formation of the ZnS shell can induce a significant red shift in the absorption and emission spectra of the NPLs, along with a broadening of the emission peak compared to the core NPLs. These effects are attributed to the relaxed vertical confinement of electrons and holes, change in the dielectric constant, as well as electron shakeup effects in negatively charged trions and confinement potential fluctuations induced by surface charges and structural dynamics.^13,50^ Therefore, to prevent excessive red-shifting of absorption/emission spectra, we grow a thin ZnS shell on the seed NPLs at this stage, ensuring that the core/thin-shell NPLs would emit in the green region. Herein, the thickness of the shell was carefully controlled by reducing the injection amount of the anion and monitoring the emission/absorption spectra of the NPLs throughout the process so that the reaction was quenched once the peak emission of the core/thin-shell NPLs reached ∼560 nm. As shown in Figure 1c, the electron-heavy hole and electron-light hole excitonic transitions of the synthesized 4 ML CdSe_0.7_S_0.3_/ZnS alloyed core/thin-shell NPLs are broadened and shifted to 522 and 460 nm, respectively, with a weak emission peak centered at 558 nm, yielding a large Stokes shift of ∼36 nm. The fwhm was 27.3 nm with a low PL-QY of <6%. Additionally, a broad trap emission is observed in the photoluminescence spectrum (Figure S2), which can be attributed to the characteristics of the thin shell. This leads to charge carrier trapping, which in turn reduces both the overall emission and the PL-QY of the NPLs.^51^

In the next step, the CdSe_0.7_S_0.3_/ZnS alloyed core/thin-shell NPLs with an average ZnS shell thickness of ∼1 ML (as determined by the TEM images and validated by the ratio of elements measured via XPS, SI) were subjected to the Cd-to-Zn CE reaction following the previously reported method with some modifications.^15^ Briefly, the CE reaction was conducted using the ZnI_2_ precursor in 1-octadecene (ODE) in the presence of oleylamine (OLA) as a ligand. A dispersion of the CdSe_0.7_S_0.3_/ZnS alloyed core/thin-shell NPLs in a mixture of ODE/TOP = 1/1 was injected into the ZnI_2_–ODE solution, and the CE process was carried out at 310 °C for several hours, with aliquots taken at various time intervals for optical measurements and monitoring the progress of the reaction. During the CE process, OLA, as a soft Lewis base, coordinates with Zn (II) ions, enhancing their solubility in organic solvents, facilitating their delivery to the NPL surface, and promoting the CE. Using ZnI_2_, a reactive Zn-OLA complex with a weak binding energy is formed that readily interacts with the NPL surface, driving the exchange of Zn (II) with Cd (II) in the NPL core.^43,52^ Moreover, OLA as an effective surface passivation agent for NPLs can enhance their PL-QY.^43^ TOP was used as the soft base to stabilize Cd (II). In addition, TOP binds to the surface of CdSe_0.7_S_0.3_/ZnS core/thin-shell NPLs, passivates the defects, and preserves the structural integrity and optical properties during the CE process. This passivation can prevent aggregation and degradation under high temperatures, ensuring uniform exchange and maintaining photoluminescence by minimizing nonradiative recombination pathways.^53^ The Cd-to-Zn CE was conducted at 310 °C due to the high CE efficiency between 300 and 320 °C.^15^ This boosts the mobility and diffusion of Zn (II) ions into the nanocrystal core, which promotes the CE reaction rate and allows for more Cd to be replaced with Zn. At this temperature, Zn (II) ions can exchange with both the near-surface and lattice Cd atoms located deeper within the NPLs core.^15^

As an example, the absorption and photoluminescence spectra of the typical blue- and green-emitting CdZnSeS/ZnS quaternary alloyed core/thin-shell NPLs synthesized through 150 and 30 min Cd-to-Zn CE on the CdSe_0.7_S_0.3_/ZnS alloyed core/thin-shell NPLs are shown in Figure 1d,h, respectively. Both sets of NPLs showed blue-shifted absorption and photoluminescence spectra compared to the seed core/thin-shell NPLs, before CE, with emission peak wavelengths centered at 473 (CE for 150 min) and 501 nm (CE for 30 min). The fwhm was 33 nm for the blue-emitting quaternary alloyed core/thin-shell NPLs and 29.7 nm for the green ones.

To closely monitor the absorption/emission spectra of the NPLs during the Cd-to-Zn CE, we conducted two batches of CE reaction for 30 and 210 min, resulting in CdZnSeS/ZnS quaternary alloyed core/thin-shell NPLs with green and blue emissions, respectively. Figure 2a,b illustrates the evolution of the absorption and photoluminescence spectra of the aliquots taken at various time intervals during the 210 min CE reaction. A detailed optical study of the first 30 min of the CE process is also provided in Figure S3. As can be seen in Figure 2a,b, the absorption and photoluminescence spectra exhibit a continuous blue shift as the CE reaction progresses over time, confirming the formation of CdZnSeS alloys in the NPL core, which has a wider bandgap than CdSeS core NPLs. From Figure 2a, the absorption profile of the CdSe_0.7_S_0.3_/ZnS core-thin-shell NPLs initially exhibits two distinct excitonic peaks. However, as the Cd-to-Zn CE reaction proceeds, these peaks broaden and become less resolved, and after 30 min of the reaction, they are no longer discernible. Here, the changes in the absorption profile of the cation-exchanged NPLs reflect the formation of CdZnSeS alloys in the NPL core. The evolution of the emission spectra in Figure 2b reveals that upon conducting the CE reaction in the NPL core, the emission peak gradually blue-shifts from 558 nm of the initial CdSe_0.7_S_0.3_/ZnS core/thin-shell NPLs toward shorter wavelengths. Here, an initial rapid blue shift was observed during the first 30 min of the CE reaction, particularly within the first 10 min, after which it slowed down. The overall spectral tuning range of ∼100 nm (from green to blue) was achieved after 210 min of CE reaction. The variations in the emission peak wavelength and corresponding fwhm of the NPLs during the CE reaction are depicted in Figure 2c, which shows that the emission peaks of the cation-exchanged NPLs gradually broaden with the fwhm gently increasing from 27.3 nm for the core/thin-shell NPLs (before CE) to 30.8 nm (with a peak position (λ_em_) at 494 nm) after 60 min, and further to 34.3 nm (λ_em_ = 460 nm) after 210 min of CE reaction. The observed broadening in the PL emission during the CE reaction process can be primarily attributed to the increasing compositional inhomogeneity within the NPL core. Additionally, a trap emission was initially observed in the 550–750 nm region of the photoluminescence spectra for the cation-exchanged NPLs (Figure S4), as a result of probable surface etching at higher temperatures and insufficient surface passivation by the thin shell. However, we noticed that the intensity of this trap emission peak gradually decreases over time and reaches its minimum level after 72 h when the colloidal solutions of the cation-exchanged NPLs are stored under ambient conditions. Moreover, increasing the thickness of the cation-exchanged NPLs through the secondary HI shell coating, the last step of the synthesis approach, effectively eliminates this trap emission.

**Figure 2:**
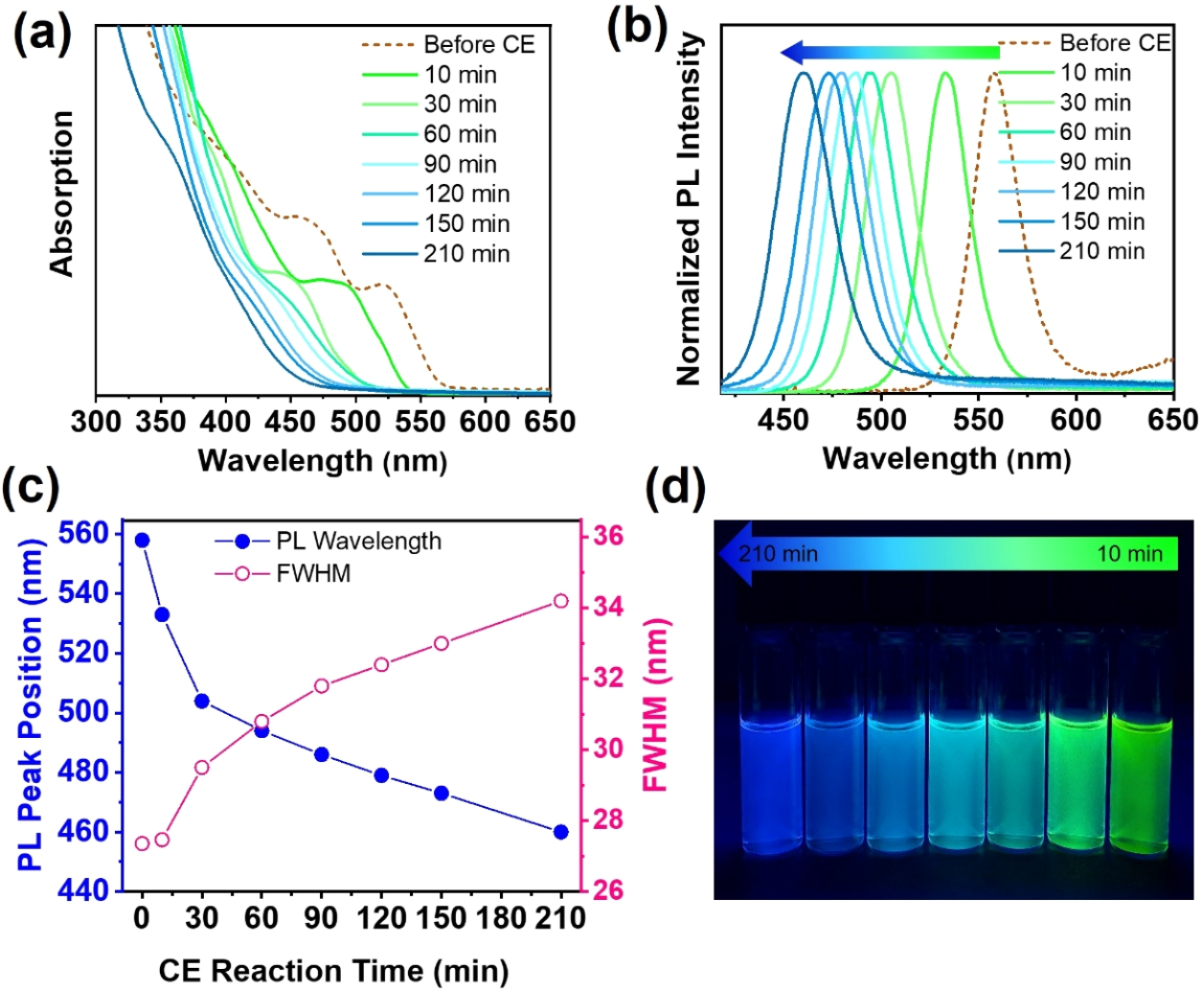
Evolution of (a) absorption spectra, (b) PL spectra, and (c) the PL peak position along with the fwhm of the 4 ML CdZnSeS/ZnS quaternary alloyed core/thin-shell NPLs taken at different time intervals of CE reaction for 210 min at 310 °C. To minimize the trap emission peak in cation-exchanged NPLs, PL/absorption spectra were recorded after storing the samples under ambient conditions for 3 days. (d) Photograph of the corresponding NPLs dispersed in hexane under 365 nm UV light.

Next, the effect of the Zn precursor concentration on the progress of the CE process was studied for 3 different concentrations of ZnI_2_, namely, 1.25 mmol for low, 1.6 mmol for medium, and 2.2 mmol for high, during a 2 h CE at 310 °C. The evolution of the absorption and photoluminescence spectra of these three sets of NPLs is presented in Figure S5a–f, and the variations in their emission peak wavelength and fwhm values as a function of CE reaction time are depicted in Figure S6a,b, respectively. From the obtained results, increasing the ZnI_2_ concentration accelerates the CE reaction, leading to a rapid blue shift in the emission peak of the NPLs, particularly during the early stages of the reaction (Figure S6a), however, higher ZnI_2_ concentrations also lead to further PL peak broadening (Figure S6b). Here, after a 2 h CE reaction, the emission peaks were blue-shifted from 558 nm (before CE) to 479 nm (with fwhm 32.4 nm), 476 nm (with fwhm 34.8 nm), and 471 nm (with fwhm 38.2 nm) for the CE core/thin-shell NPLs synthesized using low, medium, and high concentrations of ZnI_2_, respectively. Therefore, while higher dopant concentrations speed up the CE reaction, they introduce more defects and inhomogeneity, resulting in compromised optical properties. In addition, the PL-QY of these blue-emitting NPLs was measured after increasing their thickness through the HI shell coating method. As expected, a high PL-QY of 74% was obtained for the CdZnSeS/ZnS core/thick-shell NPLs synthesized with a low concentration of ZnI_2_, and it decreased to 58% and a minimum of 44% for those synthesized with medium and high concentrations of ZnI_2_, respectively.

In the final step of the synthesis, two CdZnSeS/ZnS quaternary alloyed core/thin-shell NPLs emitting in the green and blue spectral regions with emission wavelengths of 501 and 473 nm were used as the seed for the upcoming ZnS HI shell growth to improve their surface passivation and enhance the optical properties. The absorption and photoluminescence spectra of the final blue- and green-emitting thick-shelled NPLs are presented in Figure 1e,i, respectively. Notably, the thick ZnS shell growth caused only a minimal red shift in the absorption and emission spectra of the two seed NPLs. The emission peak wavelengths were centered at 475 and 509 nm for the final blue- and green-emitting thick-shelled NPLs, with only slight red shifts of 2 and 8 nm, respectively, compared with the CE alloyed core/thin-shell NPLs. The PL-QY strongly enhanced to 74 and 72% for the blue- and green-emitting NPLs, and the fwhm remained reasonably below 33.8 nm. The enhanced PL-QY of these thick-shell NPLs is likely due to the strong passivation of the surface with the thick, wide bandgap ZnS shell. The overall optical properties of the obtained NPLs through the proposed four-step synthesis procedure are summarized in Table S1. In addition, to evaluate the photochemical stability of the CdZnSeS/ZnS quaternary alloy core/thick-shell NPLs, we monitored their optical properties under continuous exposure to a 365 nm UV lamp at room temperature. For this purpose, the absorption and PL spectra of diluted solutions of NPLs in hexane were measured over time. The results are shown in Figure S7. After 6 h of UV irradiation, the shape or peak position of the absorption and PL spectra did not show significant changes, which indicates that the synthesized CdZnSeS/ZnS quaternary alloyed core/thick-shell NPLs possess excellent photochemical stability.

The structural properties of the NPLs obtained throughout each synthesis step were studied using transmission electron microscopy (TEM), X-ray diffraction (XRD), and X-ray photoelectron spectroscopy (XPS). Figure 3a–d shows the high-angle annular dark-field scanning transmission electron microscopy (HAADF-STEM) images of CdSe_0.7_S_0.3_ alloyed core NPLs, CdSe_0.7_S_0.3_/ZnS alloyed core/thin-shell NPLs, blue-emitting CdZnSeS/ZnS quaternary alloyed core/thin-shell NPLs after 150 min CE, and the final blue-emitting thick-shelled NPLs. The seed CdSe_0.7_S_0.3_ alloyed core NPLs feature rectangular shapes with a lateral size of 25.7 ± 1.5 × 9.8 ± 0.9 nm^2^ and a vertical thickness of 1.5 ± 0.1 nm. Growing the ZnS thin shell on the core NPLs increased their thickness to 2.2 ± 0.1 nm, while the subsequent Cd-to-Zn cation-exchange reaction in the core of CdSe_0.7_S_0.3_/ZnS core/thin-shell NPLs did not alter the thickness or lateral size of the NPLs. Remarkably, despite the higher reaction temperature, there was almost no change in the 2D shape and size of the NPLs after 150 min of CE reaction. The results surpass previous studies, where only 10 min of Cd-to-Zn CE at 320 °C on the CdSe/ZnS core/shell NPLs resulted in a decrease in both the short and long edge lengths of the NPLs.^15^ Finally, through the secondary HI shell coating, the thickness of the cation-exchanged NPLs increased to 3.5 ± 0.1 nm, and the final blue-emitting CdZnSeS/ZnS quaternary alloyed core/thick-shell NPLs showed well-defined and sharper edges compared to the thin-shelled ones. Here, the lateral size of the final thick-shelled NPLs is 24.1 ± 1.9 × 9.1 ± 0.7 nm^2^, showing a slight decrease along the longer edges compared to the thin-shelled NPLs, mainly due to dissolution/recrystallization processes. However, their lateral aspect (the length of the long edge to the short edge) remains nearly the same as in the thin-shelled NPLs, confirming that the overall shape was well preserved throughout the proposed synthesis approach. Comparison of the corresponding TEM-EDS mapping of Cd and Zn elements for the blue-emitting NPLs before and after the 150 min CE in Figures 3e,f and S8 clearly reveals a reduction in the Cd content and an increase in the Zn content for the cation-exchanged NPLs. In addition, the HAADF-STEM and TEM-EDS mapping images of the green-emitting NPLs before and after the 30 min CE reaction, in Figures S9 and S10, follow the same observation.

**Figure 3:**
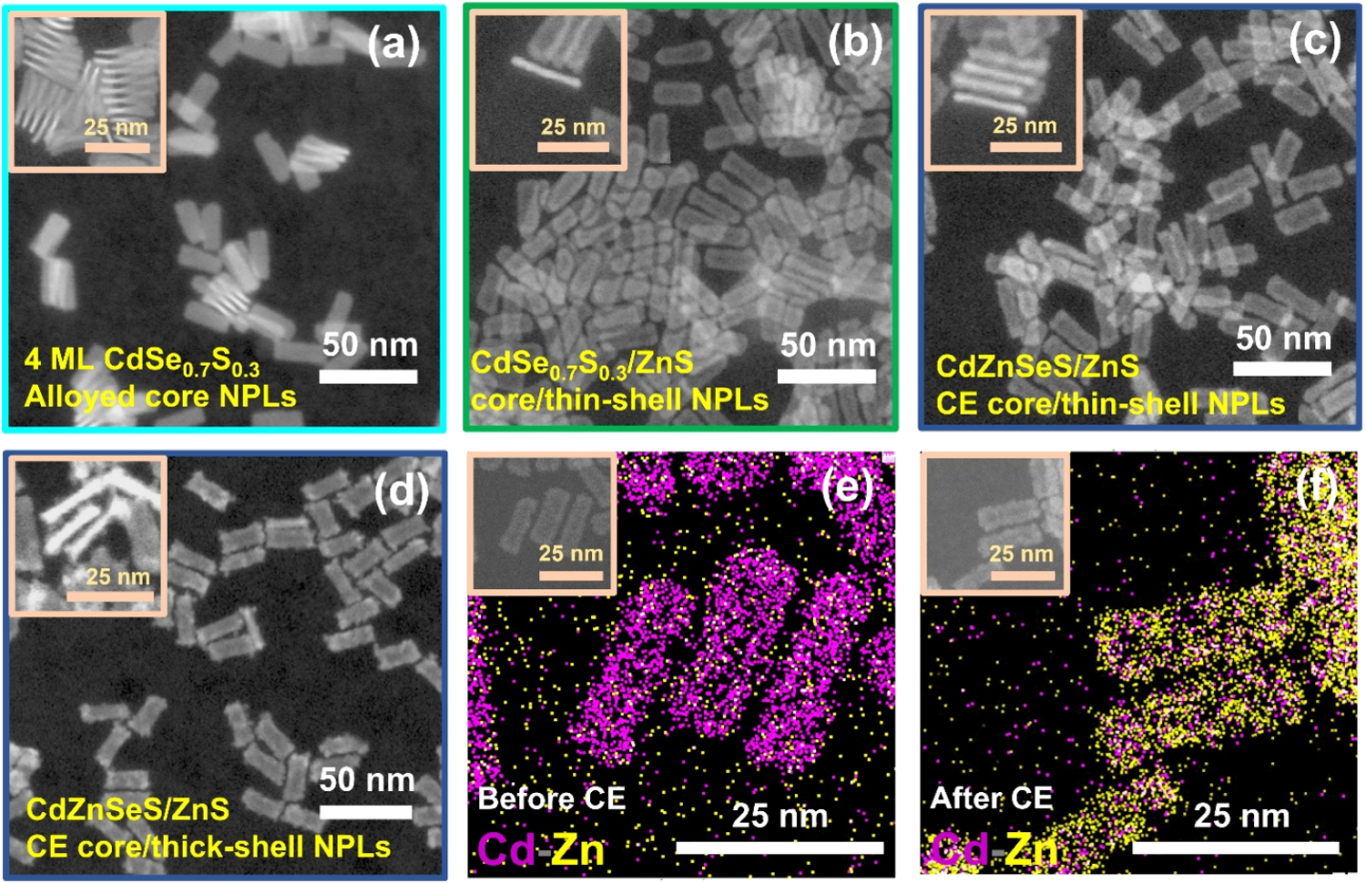
HAADF-STEM images of (a) CdSe_0.7_S_0.3_ alloyed core NPLs, (b) CdSe_0.7_S_0.3_/ZnS alloyed core/thin-shell NPLs, (c) blue-emitting CdZnSeS/ZnS quaternary alloyed core/thin-shell NPLs synthesized via 150 min CE at 310 °C, and (d) the final blue-emitting CdZnSeS/ZnS quaternary alloyed core/thick-shell NPLs. The inset images of (a–d) show the vertical thickness of the NPLs. (e and f) TEM-EDS mapping images of Cd–Zn for the blue-emitting NPLs before and after 150 min Cd-to-Zn CE reaction. The inset images of (e) and (f) show the corresponding TEM images of the NPLs.

The crystal structure of the NPLs was analyzed using XRD and is depicted in Figure 4a,b at different synthesis steps for the blue- and green-emitting NPLs, respectively, along with standard patterns for the bulk materials. The obtained XRD data of the corresponding NPLs were provided in Tables S2 and S3. The XRD patterns show that throughout the proposed synthesis approach, the NPLs maintained their zinc-blende crystal structure with distinct crystal planes of (111), (220), and (311), where the (111) planes exhibit the highest diffraction intensity across all NPLs. Upon growing the ZnS thin shell on the CdSe_0.7_S_0.3_ alloyed core, the position of the diffraction peaks is slightly shifted to higher degrees, due to the smaller lattice constant of ZnS.^32^ Following the Cd-to-Zn CE reaction in the core of NPLs and the subsequent growth of the final ZnS shell, further peak shifts to larger angles are noted. Since no new diffraction peaks are observed in the XRD patterns of the studied NPLs, the possibility of renucleation or the formation of different subspecies is significantly minimized. As presented in Tables S2 and S3, the width of the (111) reflection decreases as the NPLs transition from the CdSe_0.7_S_0.3_ alloyed core structure to the final CdZnSeS/ZnS quaternary alloyed core/thick-shell configuration. This peak narrowing indicates improved crystallinity attributed to the epitaxial growth of the ZnS shell and an increase in NPL thickness.^14^ Furthermore, the interlayer space (d) of all of the planes slightly decreases from the core to the final thick-shelled NPLs.

**Figure 4:**
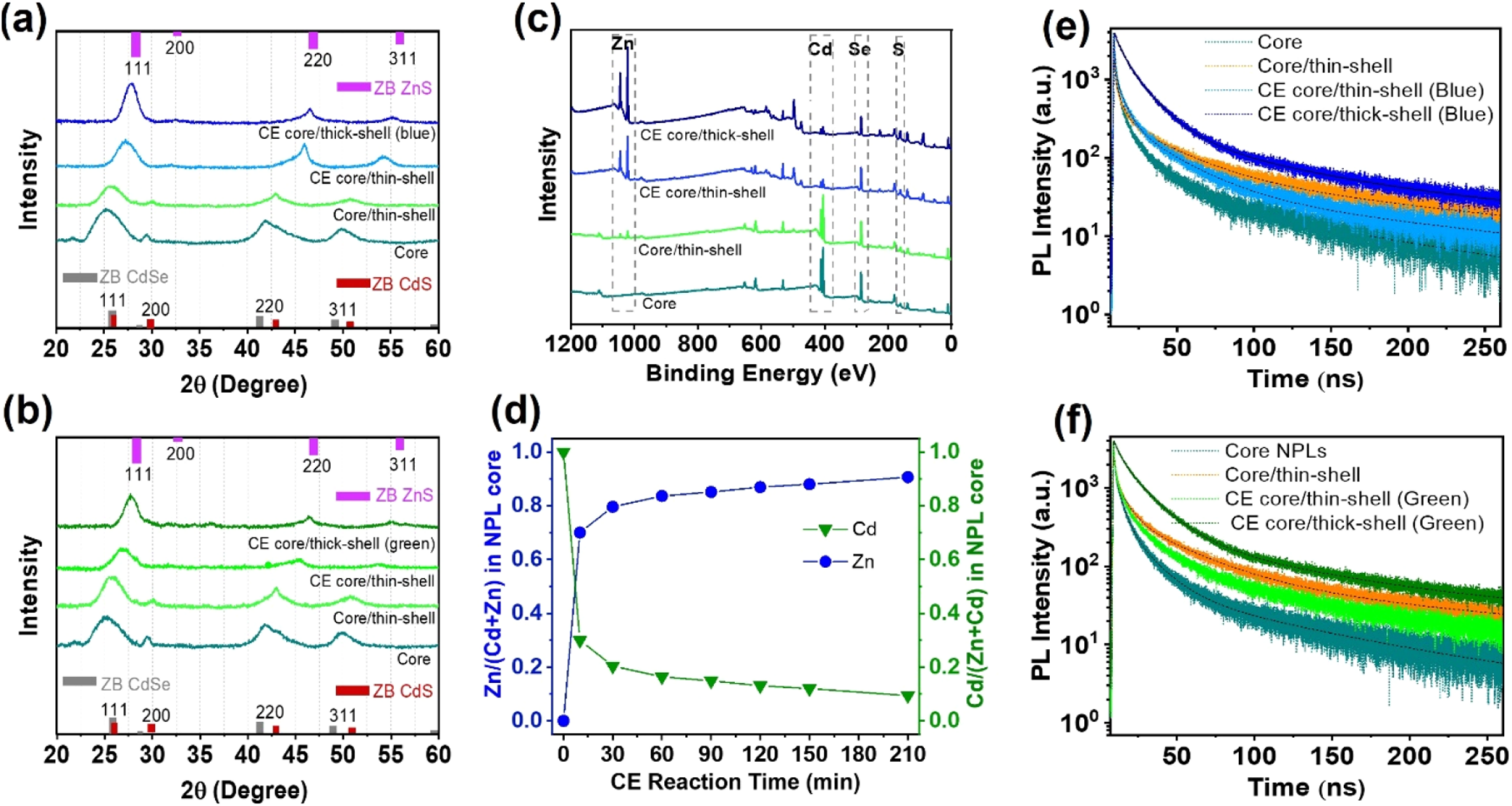
(a, b) XRD patterns of the CdSe_0.7_S_0.3_ alloyed core, CdSe_0.7_S_0.3_/ZnS alloyed core/thin-shell, CdZnSeS/ZnS quaternary alloyed core/thin-shell, and the final CdZnSeS/ZnS quaternary alloyed core/thick-shell for the blue-emitting NPLs (first row), and green-emitting NPLs (second row). (c) XPS survey spectra of the same samples for the blue-emitting NPLs. (d) Zn (blue circles) and Cd (green triangles) contents in the core of cation-exchanged NPLs as a function of reaction time. (e, f) TRF curves of the same samples for the blue-emitting NPLs (first row) and green-emitting NPLs (second row). The CE reaction was conducted for 150 and 30 min for the blue- and green-emitting NPLs, respectively.

The elemental composition of the synthesized NPLs was studied by XPS. Figures 4c and S11 present the XPS survey spectra at different synthesis steps for the blue- and green-emitting NPLs, respectively, and the corresponding atomic percentages of elements are given in Table S4. Moreover, the resolved XPS spectra of Cd 3d, Zn 2p, Se 3d, and S 2p peaks for the blue- and green-emitting NPLs before and after CE are given in Figure S12. Herein, the XPS elemental composition studies revealed that the Cd content of the final blue- and green-emitting CdZnSeS/ZnS quaternary alloyed core/thick-shell NPLs decreased by 96.5% and 94% (with respect to the total cation content), respectively, compared to the core-only NPLs, while their Zn content increased, which further confirms successful Cd-to-Zn exchange and the formation of the ZnS shell. Furthermore, based on the XPS elemental composition results (Table S4), the number of ZnS shell layers in the final blue-emitting quaternary alloyed core/thick-shell NPLs was evaluated to be ca. 3–4, with 1–2 layers deposited during the thin-shell growth step on the CdSe_0.7_S_0.3_ core NPLs.

XPS analysis was also used to examine the variations in Cd and Zn ratios in the core of the CdZnSeS/ZnS quaternary alloyed core/thin-shell NPLs during the 210 min Cd-to-Zn CE reaction at 310 °C. Here, by evaluating the XPS elemental compositions of the NPLs before and after the CE reaction and considering the fact that each core or shell layer has the same volume and same amount of cations originated from their 2D planar geometry, we were able to calculate the Cd and the Zn ratios in the core of CdZnSeS/ZnS quaternary alloyed core/thin-shell NPLs. Figure 4d depicts the changes in the Zn and Cd ratios in the NPL core after different CE reaction times, with the obtained data provided in Table S5. Given the temperature of the CE reaction (310 °C), which is above the alloying point of ZnSe and CdSe, the Cd-to-Zn exchange is expected to proceed more effectively throughout the entire CdSeS core both through the exposed Cd atoms at the side surface and by diffusion of Zn atoms into the NPL core and subsequent exchange with Cd.^15^ As shown in Figure 4d and Table S5, the Cd-to-Zn exchange reaction occurred rapidly during the first 10 min with the most significant compositional change, where 70% of Cd was replaced with Zn in the NPLs core. Then, the Cd-to-Zn exchange percentage reached ∼80% after 30 min, and further ∼88% after 150 min CE reaction. Consequently, the rate of the CE reaction slowed notably after the first 30 min. In the early stages of the CE reaction, the Cd atoms on or near the surface of the core are more easily accessible, leading to a rapid exchange with Zn. As the CE reaction continues, the remaining Cd atoms are located deeper within the core, making them less accessible. This necessitates the diffusion of Zn cations further into the NPL core to replace those Cd atoms, which slows down the reaction rate and can also be the reason for the considerable but gradual blue shift in the emission peak of the NPLs after the initial 10 min of CE process, although 70% of Cd was replaced by Zn during the first 10 min. Additionally, as more Cd is replaced by Zn, the driving force for the exchange, which is the elemental gradient between the Cd-rich and Zn-rich reaction zones, decreases. Initially, there is a high chemical potential difference between Cd and Zn in the core, which drives the rapid exchange. As the exchange reaction progresses and the core composition changes, this potential difference decreases, resulting in a slower CE reaction rate. Besides these, as the CE reaction progresses, the reactivity of the remaining Cd might decrease due to changes in the local environment.

In addition to the spectral shift of the emission peak wavelength, cation exchange in semiconductor nanocrystals is known to significantly affect the recombination dynamics. To understand the underlying reason for the PL-QY enhancement of the final CdZnSeS/ZnS quaternary alloy core/thick-shell NPLs, the recombination dynamics were investigated via time-resolved fluorescence (TRF) spectroscopy. Figure 4e,f displays the fluorescence decay curves at different synthesis steps for the blue- and green-emitting NPLs, respectively, with the TRF decay components summarized in Tables S6 and S7. Each fluorescence decay curve was fitted with a three-exponential function. Growing the ZnS thin shell on the CdSe_0.7_S_0.3_ alloyed core NPLs increased the intensity-averaged fluorescence lifetime (τ_int_) from 34.2 to 62.7 ns due to the surface passivation of the core by shell coating, relaxed confinement, which also increases the electron and hole distance, and change in the dielectric environment around the core. Conducting the 150 min Cd-to-Zn CE reaction in the core of NPLs decreased the τ_int_ to 46.9 ns for the blue-emitting CdZnSeS/ZnS quaternary alloy core/thin-shell NPLs. Similarly, the green-emitting quaternary alloyed core/thin-shell NPLs followed a similar trend, exhibiting an ∼9 ns decrease in τ_int_ after 30 min CE. The CE process alters the core composition, forming CdZnSeS alloy with a wider bandgap which results in faster recombination rates and shorter lifetimes. Also, the incorporation of Zn during the CE reaction may lead to the formation of defects such as Zn-related traps or lattice strain. These defects can act as nonradiative recombination centers, hence leading to shorter PL lifetimes. Finally, increasing the thickness of the quaternary alloyed core/thin-shell NPLs through the secondary HI shell coating led to a significant increase in τ_int_, reaching ∼78 ns for the blue-emitting CdZnSeS/ZnS quaternary alloyed core/thick-shell NPLs, with PL-QY of 74%, and ∼71 ns for the green-emitting ones, with PL-QY of 72%. This increase is primarily due to the relaxed quantum confinement leading to increased distance between electron and hole and enhancement in PL-QY as a result of passivation of defect sites, which prolongs the recombination lifetime.^13,54^

Motivated by the high PL-QY of the synthesized CdZnSeS/ZnS quaternary alloy core/thick-shell NPLs through our proposed synthetic approach, we fabricated and investigated high-performance blue-emitting NPL-LEDs based on these NPLs. These engineered CdZnSeS/ZnS quaternary alloyed core/thick-shell NPLs are highly promising candidates as an emissive layer for LEDs owing to their reduced reabsorption losses and suppressed fluorescence resonance energy transfer (FRET) as a result of their highly efficient Stokes shifted emission. Blue-emitting CdZnSeS/ZnS quaternary alloy core/thick-shell NPLs with a PL peak at 475 nm and a PL-QY of 74% were used as the emitting layer. The fabricated LEDs consist of a transparent indium tin oxide (ITO) cathode, followed by a ZnMgO electron transport layer (ETL), a CQW-based emitting layer, a CBP hole injection layer (HIL), a MoO_3_ hole transport layer (HTL), and an Al anode (detailed information on the device fabrication is provided in the Methods). The schematic of the structure and energy band diagram of the fabricated NPL-LED are shown in Figure 5a,b. In this LED structure, we used CBP as the hole injection layer owing to its high hole mobility and relatively large energy offset with our NPLs which ensure the confinement of electrons within the active layer, and we employed Mg-doped ZnO rather than ZnO to suppress the exciton quenching as a result of nonradiative energy transfer^55,56^ and hence to maximize the efficiency. A cross-sectional HAADF-STEM image of the fabricated LED device (Figure 5c) clearly shows each deposited layer. The thickness of the spin-coated CQW layer is ∼28 nm, and the NPLs in this layer are almost in perfect face-down orientation as can be seen in the cross-sectional image. Such an orientation of two-dimensional NPLs in film helps to improve the out-coupling efficiency and hence the EQE of the device.

**Figure 5:**
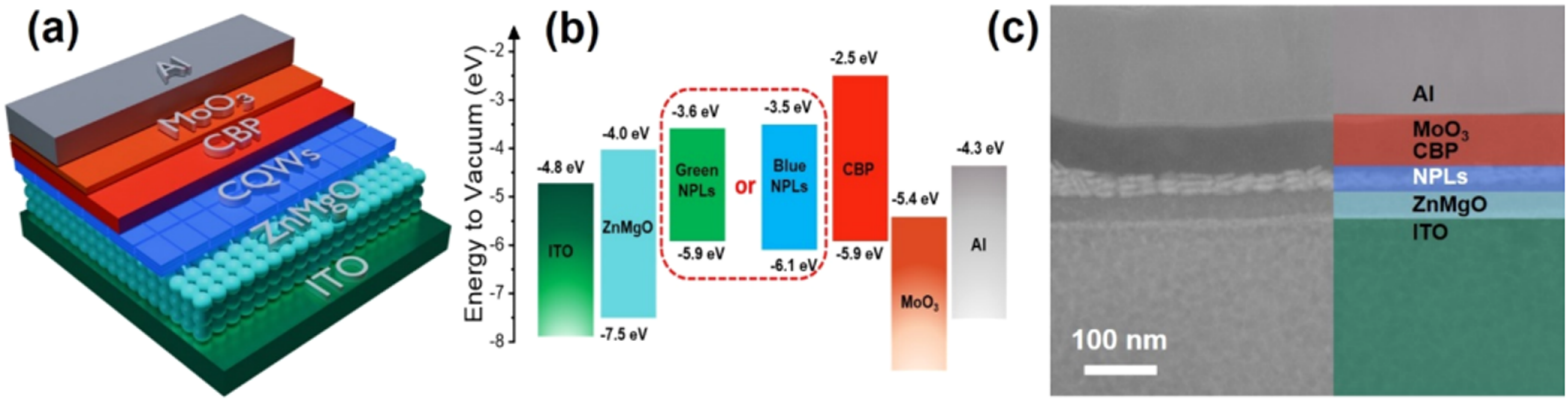
(a) Schematic representation of the NPL-LED structure with blue-emitting CdZnSeS/ZnS quaternary alloyed core/thick-shell NPLs as the emitting layer, (b) flat band energy level diagram of each layer deposited in the NPL-LED device, and (c) cross-sectional HAADF-STEM image of the device showing the layers with different colors.

The performance and characteristics of the fabricated NPL-LEDs are shown in Figure 6. Our blue-emitting NPL-LEDs achieved an exceptional external quantum efficiency (EQE) of 11.3% (Figure 6a), which is the highest reported value to date for the blue-emitting NPL-LEDs.^15,18,38,42,44,45^ In addition, our blue LED exhibits a low turn-on voltage of 2.5 V, and at 1000 cd/m^2^, the voltage is lower than 3 V. This is lower than the voltages reported in the literature for other NPL-based LEDs at the same luminance level,^15,18,38,42^ which can be attributed to the efficient charge injection in our LED device. Further, our LED shows a high maximum luminance of 12,451 cd/m^2^ (Figure 6b), which is comparable to the best results from blue NPL-LEDs.^15,45^ Figure 6c presents the typical EL spectrum of the blue-emitting NPL-LED device, showcasing bright EL with a peak located at 482 nm and high color purity at operating voltages up to 7 V with Commission Internationale de L’Eclairage (CIE) coordinates of (0.1004, 0.2166) (Figure 6d). Notably, our blue NPL-LED exhibits very stable EL spectra under different operating voltages, as can be seen in Figure 6c, which is a crucial parameter for practical applications. Hence, our blue LED having significantly better performance compared to the previously fabricated blue NPL-LEDs (Table S8) addresses the longstanding performance limitations of the blue NPL-LEDs, which have historically lagged behind their red-emitting counterparts.

**Figure 6:**
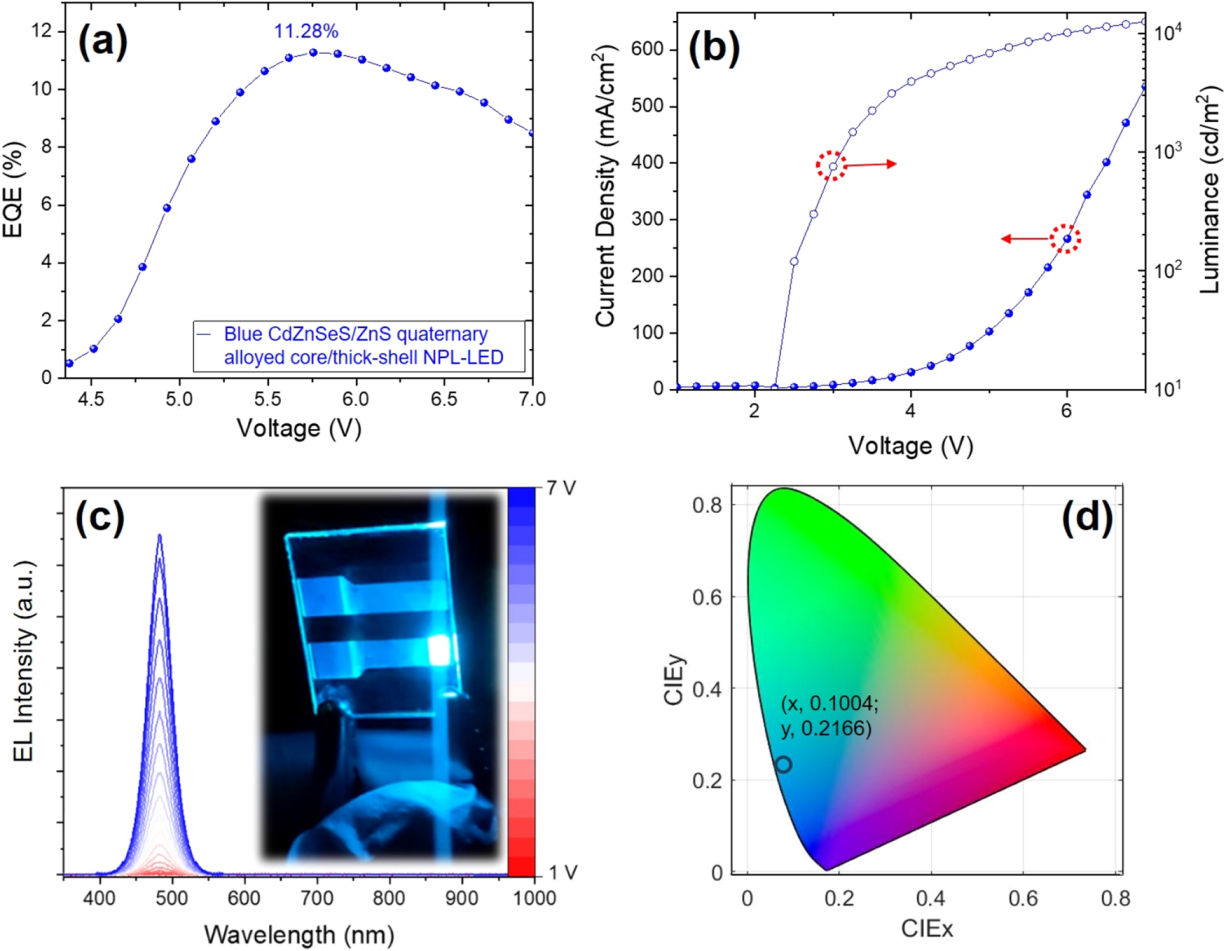
(a) EQE vs driving voltage, (b) current density–luminance–voltage characteristics, (c) EL spectra under different voltages (the inset image shows the fabricated NPL-LEDs at 7 V), and (d) CIE gamut. All for the blue-emitting NPL-LED with CdZnSeS/ZnS quaternary alloyed core/thick-shell NPLs as the active material.

In addition, we fabricated green NPL-LEDs using the same device structure with green-emitting CdZnSeS/ZnS quaternary alloy core/thick-shell NPLs as the emitting layer. The performance and characteristics of the fabricated NPL-LEDs are given in Figure S13. A typical fabricated green NPL-LED working at 7 V, which emits at 516 nm with Commission Internationale de L’Eclairage (CIE) coordinates of (0.19, 0.64), is also shown in Figure S13. This green NPL-LED exhibits an EQE of 6.3% and a maximum luminance of 13,999 cd/m^2^ with a low turn-on voltage of 2.7 V. The EQE, luminance, and turn-on voltage of our developed green NPL-LED are comparable to the best reported values for NPL-based green LEDs.^13,15,57–59^

## Conclusions

In conclusion, we demonstrated the synthesis of highly efficient blue- and green-emitting CdZnSeS/ZnS quaternary alloyed core/shell colloidal NPLs with controllable shell thickness and core composition, which allows for bandgap engineering and hence tuning of the emission wavelength. Introducing Zn atoms into the CdSe_0.7_S_0.3_ alloyed core through the CE process and successfully passivating the structure with a thick ZnS shell through the two-step HI shell coating allowed us to achieve highly efficient blue or green PL emissions, depending on the core composition, while the 2D shape and lateral size of the NPLs were well preserved throughout the proposed synthetic approach. Notably, only a minimal PL red shift was observed after HI shell thickening of the cation-exchanged NPLs, which is a significant advantage for suppressing nonradiative Auger recombination, which decreases as the volume of the emitter increases, while keeping the emission color, unlike other core/shell NPLs, in which the emission color significantly red-shifts (>100 nm) during the shell growth.^60^ The resulting thick-shelled NPLs were trap-free and exhibited enhanced PL-QY of >70% and τ_int_ ∼105 ns for both the blue- and green-emitting NPLs as well as Stokes shift of more than 40 nm. This innovative approach enabled blue-emitting CdZnSeS/ZnS quaternary alloyed core/thick-shell NPL-based LED devices to significantly address the longstanding performance limitations of blue NPL-LEDs, which have historically lagged behind their red-emitting counterparts. The fabricated blue NPL-LED device with the CdZnSeS/ZnS quaternary alloyed core/thick-shell NPLs (PL 475 nm) as the active material, exhibits a record EQE of 11.3% with a low *V*_T_ of <2.5 V and *L*_max_ of 12,451 cd/m^2^, which represents a significant advancement in blue NPL-LEDs compared to most previous reports.^15,18,38,42,44^ Our proposed approach marks a turning point in addressing the challenges associated with fabricating high-efficiency blue-emitting CdSe-based core/shell NPLs that is crucial to meet the growing demand for efficient, stable, and tunable blue light sources in advanced optoelectronic devices, especially for lasers and LEDs.

## Materials and Methods

### Chemicals

Cadmium acetate dihydrate (Cd-(OAc)_2_·2H_2_O, >98%), sodium myristate (>99%), cadmium nitrate tetrahydrate (Cd­(NO_3_)_2_·4H_2_O, 99.99%), zinc acetate (Zn­(OAc)_2_, 99.99%), zinc acetate dihydrate (≥99.0%), magnesium acetate tetrahydrate (≥99.0%), selenium (99.99%), sulfur (99.998%), zinc iodide anhydrous (99.99%), trioctylphosphine (TOP, 97%), 1-octanethiol (≥98.5%), technical-grade 1-octadecene (ODE, 90%), technical-grade oleic acid (OA, 90%), technical-grade oleylamine (OLA, 70%), ammonium sulfide solution ((NH_4_)_2_S, 40–48 wt % in H_2_O), tetramethylammonium hydroxide ((CH_3_)_4_N­(OH), 98%), ethanolamine (≥99.0%), *n*-hexane (≥97.0%), *N*-methylformamide (NMF, 99%), toluene (≥99.5%), ethanol (absolute), methanol (≥99.7%), acetone (99%), dimethyl sulfoxide (DMSO, 99%), 4,4′-bis­(N-carbazolyl)-1,1′-biphenyl (CBP), and molybdenum trioxide (MoO_3_) were obtained from Sigma-Aldrich and used without any further change.

### Synthesis of Cadmium Myristate

Cadmium myristate was synthesized according to the previously reported procedure with minor changes.^61^ Initially, two separate solutions were prepared: cadmium nitrate tetrahydrate (2.46 g in 80 mL of ethanol) and sodium myristate (6.26 g in 50 mL of ethanol). The as-prepared solutions were then mixed and stirred for 4 h to ensure a complete exchange reaction. Subsequently, the bulky solution of cadmium myristate was collected by filtration, washed three times with methanol to completely remove excess precursors, and finally vacuum-dried overnight to give cadmium myristate as a white powder. The product was stored in a refrigerator until use.

### Synthesis of 4 ML CdSe_0.7_S_0.3_ Alloyed Core NPLs

The alloyed 4 ML CdSe_0.7_S_0.3_ NPLs were synthesized following the published recipe of our group with some modifications.^35^ 340 mg of cadmium myristate, 20 mg of Se, and 30 mL of ODE were mixed in a 100 mL three-neck flask and heated to 95 °C under vacuum. The reaction mixture was degassed at 95 °C for 1 h to completely remove water and any other remaining volatile solvents. Subsequently, the temperature was increased to 240 °C, under nitrogen flow, and at 100 °C, a sulfur precursor (S/ODE, 0.2 M) was injected swiftly. When the temperature reached ∼195 °C, 120 mg of cadmium acetate dihydrate was rapidly added. The reaction was maintained at high temperature for 7 min, and then 1 mL of OA was injected into the solution while the flask was quickly quenched in a water bath. Below 70 °C, 10 mL of hexane was injected for better dissolution of the NPLs. The 4 ML CdSe_0.7_S_0.3_ NPLs were separated from undesired byproducts by the addition of ethanol and centrifugation at 6000 rpm for 6 min. The final NPLs were redispersed in hexane and kept for further use.

### Synthesis of 4 ML CdSe_0.7_S_0.3_/ZnS Alloyed Core/Thin-Shell NPLs

The CdSe_0.7_S_0.3_/ZnS core/thin-shell NPLs were synthesized via hot injection (HI) shell growth using the literature procedure with some modifications.^19^ 73 mg of zinc acetate (0.4 mmol), 6.5 mL of ODE, 1 mL of OA, and a proper amount of 4 ML CdSe_0.7_S_0.3_ core NPLs were mixed and degassed at room temperature for 1 h, and at 80 °C for 30 min. The flask was then flushed with N_2_ gas, 1 mL of OLA was added, and the temperature was set to 300 °C. At 160 °C, a sulfur precursor of 1-octanethiol in ODE (0.1 M) was started to be injected into the solution with an initial injection rate of 4 mL/h for temperatures below 240 °C and later 1 mL/h for temperatures above 240 °C. The reaction was quenched once the peak emission of the NPLs reached ∼560 nm. Then, the solution was diluted with hexane and centrifuged. The NPLs were then precipitated by the addition of ethanol and centrifugation. Finally, the CdSe_0.7_S_0.3_/ZnS core/thin shell NPLs were redispersed in hexane and kept for further use.

### Cd-to Zn Cation-Exchange Reaction on the 4 ML CdSe_0.7_S_0.3_/ZnS Alloyed Core/Thin-Shell NPLs

The 4 ML CdZnSeS/ZnS quaternary alloyed core–shell NPLs were synthesized by performing a Cd-to-Zn CE reaction on the CdSe_0.7_S_0.3_/ZnS alloyed core/thin-shell NPLs. Cd-to-Zn CE reaction was conducted following the literature procedures with minor modifications.^15^ First, 1.25 mmol of ZnI_2_, 1.25 mmol of OLA, and 2 mL of ODE were mixed and degassed at 95 °C for 30 min. The flask was then flushed with N_2_ gas, and the temperature was set to 310 °C. Meanwhile, the dispersion of a proper amount of CdSe_0.7_S_0.3_/ZnS core/thin-shell NPLs in ODE/TOP: 1/1 was added swiftly into the reaction flask. Different samples were taken from the reaction mixture at different time intervals to monitor the reaction progress by checking their absorbances and PL spectra. After completion, the reaction flask was quickly quenched in a water bath, and 2 mL of OA was injected into the flask at 80 °C. The solution was diluted with hexane and centrifuged to remove the unstable and unwanted particles. The NPLs were precipitated by the addition of ethanol and centrifugation. Finally, the resulting CdZnSeS/ZnS quaternary alloyed core/thin shell NPLs were redispersed in hexane and stored for later use.

### Synthesis of 4 ML CdZnSeS/ZnS Quaternary Alloyed Core/Thick-Shell NPLs

The 4 ML CdZnSeS/ZnS quaternary alloyed core/thin-shell NPLs were used as a seed for the final ZnS HI shell growth to increase the thickness of the NPLs. The whole procedure is similar to thin-shell growth except that the injected rate and amount of the anion precursor were changed. Herein, when the temperature reached 160 °C, 4 mL of sulfur precursor of 1-octanethiol in ODE (0.1 M) was started to be injected with an initial injection rate of 10 mL/h, and at 240 °C, the injection rate was decreased to 4 mL/h until all precursor was injected into the reaction mixture. The resulting CdZnSeS/ZnS quaternary alloy core/thick-shell NPLs were redispersed in hexane and kept for further use.

### Synthesis of ZnMgO

ZnMgO was synthesized according to the literature procedure with slight modifications.^62^ Zinc acetate dihydrate (2.95 mmol) and magnesium acetate tetrahydrate (0.05 mmol) were dissolved in 30 mL of DMSO with vigorous stirring. Then, a mixture of 5.5 mmol of tetramethylammonium hydroxide in 5 mL of ethanol was injected into the above solution at a rate of 40 mL/h. The mixture was stirred for 1 day under ambient conditions. The Zn_0.95_Mg_0.05_O nanocrystals were precipitated by adding ethyl acetate and redispersed in ethanol. To improve the solubility of Zn_0.95_Mg_0.05_O nanoparticles, inside the nitrogen-filled glovebox, 180 μL of ethanolamine was added to the mixture and then stirred for 2 h. Finally, the obtained Zn_0.95_Mg_0.05_O nanoparticles were washed with ethyl acetate and redispersed in ethanol.

### Device Fabrication and Characterization

The NPL-LEDs were fabricated on patterned indium tin oxide (ITO)-coated substrates. The substrates were first cleaned with detergent, acetone, distilled water, and 2-propanol, in sequence, for 10 min each. Then, ITO-coated substrates were treated with ozone plasma for 15 min with 30 W to eliminate the residues on the ITO surface and increase the work function of ITO. For the deposition of the electron transport layer (ETL), a solution of ZnMgO nanoparticles in ethanol with a concentration of 25 mg/mL (filtered through a 0.25 μm PTFE membrane filter) was spin-coated onto the ITO substrates at 3000 rpm for 60 s and baked at 110 °C for 30 min (thickness of 36 nm). The CdZnSeS/ZnS quaternary alloyed core/thick-shell NPLs were deposited on the ZnMgO-coated substrates via the spin-coating technique, serving as an emissive layer (thickness of 28 nm). Then, CBP, the hole injection layer (HIL) with a thickness of 62 nm, and MoO_3_, the hole transport layer (HTL), with a thickness of 5 nm, were sequentially deposited using thermal evaporation. Finally, the top Al anode layer (thickness of 165 nm) was thermally deposited under a base pressure of 1 × 10^–8^ Pa.

Current density–voltage–luminance (*J–V–L*) characteristics of NPL-LEDs were determined using an Agilent Technologies (U3606A) electrometer. The EQE measurements were performed using an integrated sphere (Newport 5.3″), which was coupled to a spectrometer (Ocean Optics QEPro).

### NPLs Characterization

Optical absorption and photoluminescence spectra of NPLs were taken from their diluted solutions in hexane using quartz cuvettes and recorded using an Agilent Cary 60 UV–vis spectrophotometer and a Varian Cary Eclipse at an excitation wavelength of 400 nm, respectively. PL-QY was measured using an integrating sphere with an excitation wavelength of 400 nm by following the method developed by de Mello et al.^63^ Time-resolved photoluminescence (TRF) measurements were obtained using the Pico Quant FluoTime 200 with an excitation wavelength of 375 nm, a pulse width of 230 ps, and a repetition rate of 2.5 MHz. The decays were fitted using FluoFit software in deconvolution mode with multiexponential decays. X-ray diffraction patterns were obtained by using the film samples of the NPLs prepared by drop-casting the NPLs solution and evaporating the solvent. Before drop-casting, the NPLs were washed two times through centrifugation in hexane/ethanol and redispersed in hexane. The measurements were taken by an X-ray diffractometer (XRD, X’Pert pro MPD, PANalytical Empyrean) equipped with a Cu Kα irradiation source (40 kV, 45 mA, λ = 1.5405 Å). Chemical and elemental analyses of the NPLs were determined by using X-ray photoelectron spectroscopy (XPS) (Thermo Fisher Scientific). For this measurement, a thin film of NPLs was coated onto the silicon wafer by using the drop-casting method and then dried under ambient conditions. High-resolution spectra were taken at a fixed pass energy of 30 eV, spot size of ∼400 μm, and step size of 0.1 eV. Transmission electron microscopy (TEM) imaging was done with an FEI Tecnai G2 Spirit BioTwin CTEM operated at 300 kV in the high-angle annular dark-field scanning transmission electron microscopy (HAADF-STEM) configuration. Focused ion beam (FIB, FEI NovaLab 600i) milling was used to prepare a thin film of the NPL-LED for TEM cross-sectional inspections.

## Supplementary Material



## Notes

Detailed XRD, XPS, and TRF characterization of CdSe_0.7_S_0.3_ alloyed core, CdSe_0.7_S_0.3_/ZnS alloyed core/thin-shell, CdZnSeS quaternary alloyed core/thin-shell, and the final thick-shelled NPLs for both the blue- and green-emitting NPLs; HAADF-STEM images of the green-emitting NPLs before and after CE; TEM-EDS mapping images of the NPLs; evaluation of photochemical stability of the final quaternary core/thick-shelled NPLs; performance and characteristics of the fabricated green NPL-LED based on green-emitting CdZnSeS/ZnS quaternary alloyed core/thick-shell NPLs; and comparison table of device performance of various blue/green LEDs (PDF)
