# Supplementary material for: Bandgap-Engineered High-Efficiency Blue- and Green-Emitting CdZnSeS/ZnS Quaternary Alloyed Core/Shell Colloidal Nanoplatelets for High-Performance Light-Emitting Devices
Source: ACS Appl Mater Interfaces. 2025 Jun 2;17(23):34206–18. doi: 10.1021/acsami.5c04630 (PMC12163922; doi:10.1021/acsami.5c04630)
Supplement: Supplementary file 1 [file am5c04630_si_001.pdf]

Supporting Information

Bandgap Engineered High-Efficiency Blue- and Green-Emitting CdZnSeS/ZnS Quaternary Alloyed Core/Shell Colloidal Nanoplatelets for High-Performance Light-Emitting Devices

Aisan Khaligh[a], Savas Delikanli[a, b], Betul Canimkurbey[a, c], Farzan Shabani[a], Furkan Isik[a], and Hilmi Volkan Demir[a, b,*]

[a] *UNAM-Institute of Materials Science and Nanotechnology and the National Nanotechnology Research Center, Department of Electrical and Electronics Engineering, Department of Physics, Bilkent University, Ankara 06800, Turkey.*

[b] *LUMINOUS! Centre of Excellence for Semiconductor Lighting and Displays, The Photonics Institute, School of Electrical and Electronic Engineering, School of Physical and Mathematical Sciences, School of Materials Science and Engineering, Nanyang Technological University, 639798, Singapore.*

[c] *Department of Physics, Polatlı Faculty of Science and Letters, Ankara Hacı Bayram Veli University, Ankara 06900, Turkey.*

* **Corresponding Author:** Prof. Hilmi Volkan Demir

E-mail: volkan@stanfordalumni.org

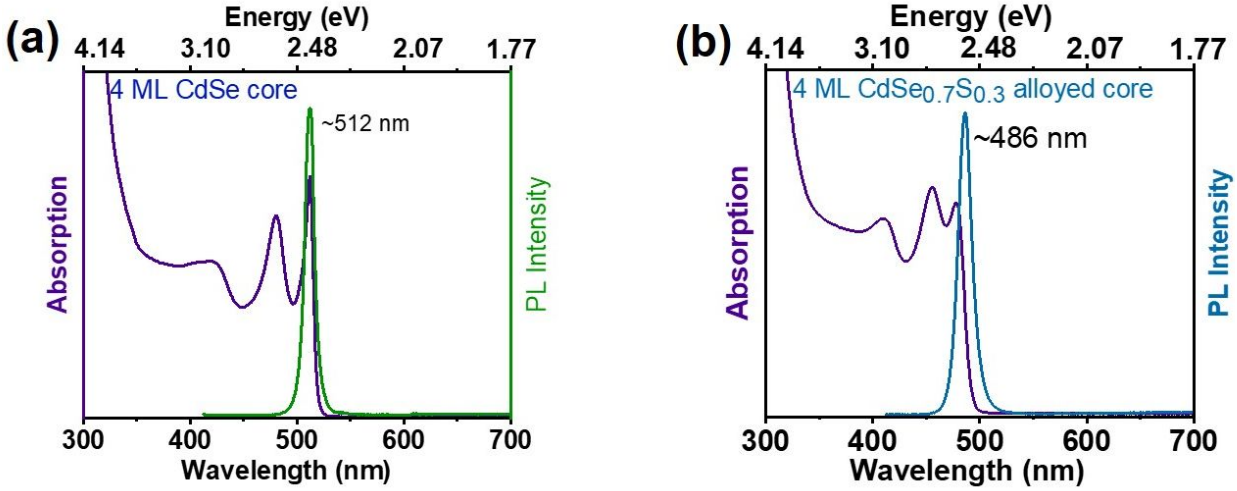

Figure S1. Absorption and photoluminescence spectra of (a) 4 ML CdSe NPLs, and (b) 4 ML $CdSe_{0.7}S_{0.3}$ NPLs.

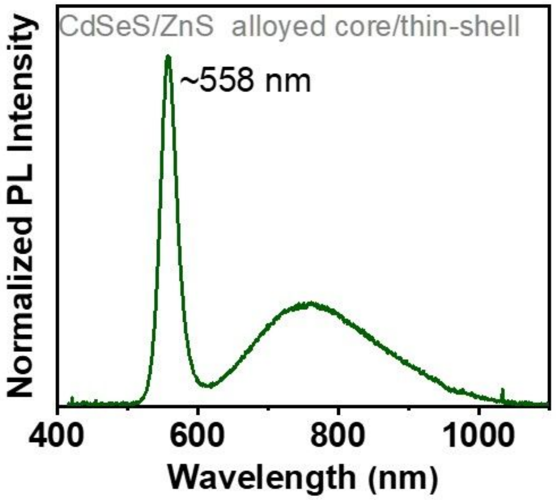

Figure S2. Photoluminescence spectrum of CdSeS/ZnS alloyed core/thin-shell NPLs, showing a trap emission peak in the range of 600-1000 nm.

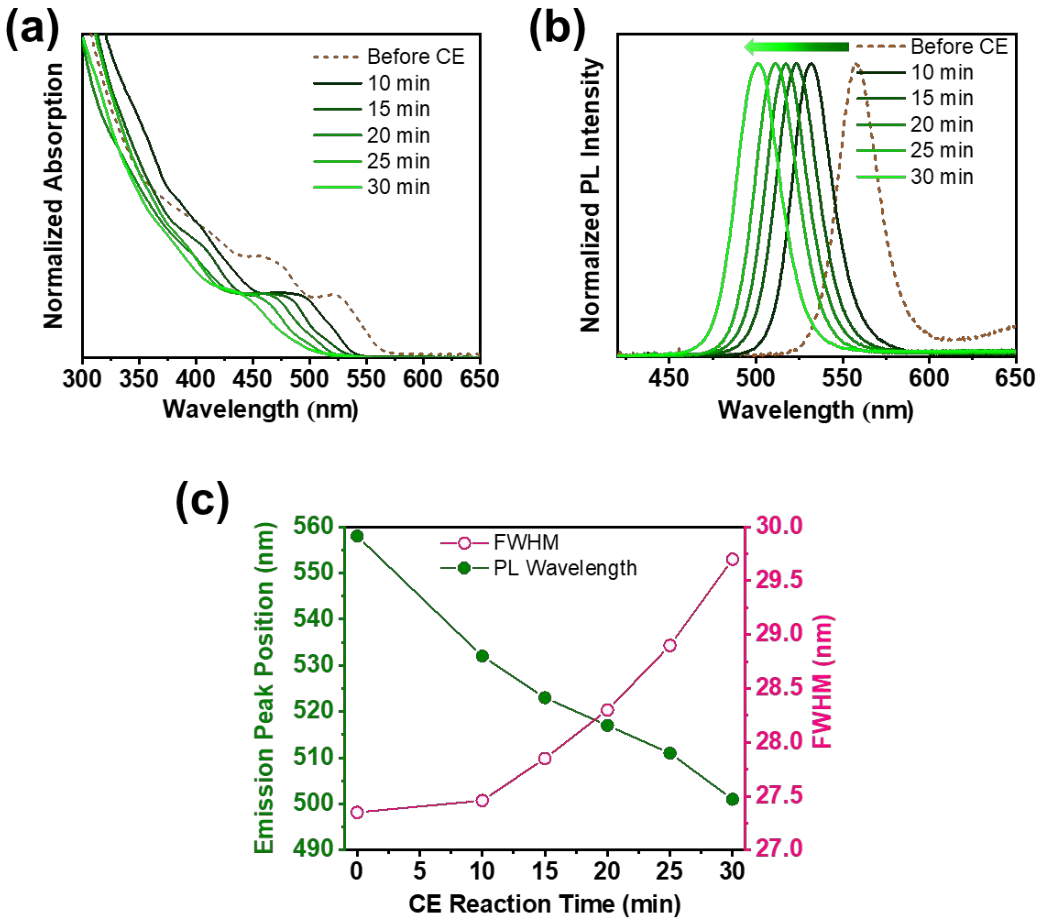

Figure S3. Evolution of the (a) photoluminescence spectra, (b) absorption spectra, and (c) the emission peak wavelength together with the FWHM of the 4 ML CdZnSeS/ZnS quaternary alloyed core/thin-shell NPLs taken at different time intervals during the 30 min Cd-to-Zn CE reaction.

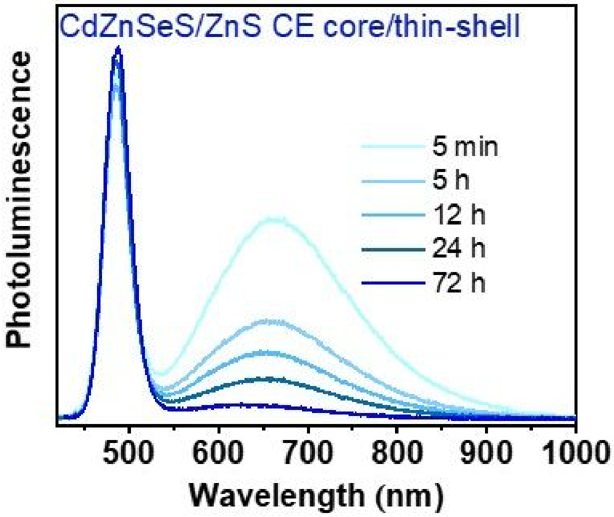

Figure S4. Decrease in the trap emission intensity of the photoluminescence spectra over time for the CdZnSeS/ZnS quaternary alloyed core/thin-shell NPLs synthesized through 90 min CE reaction. We measured the PL of the synthesized NPLs at different time intervals during storing them under ambient conditions.

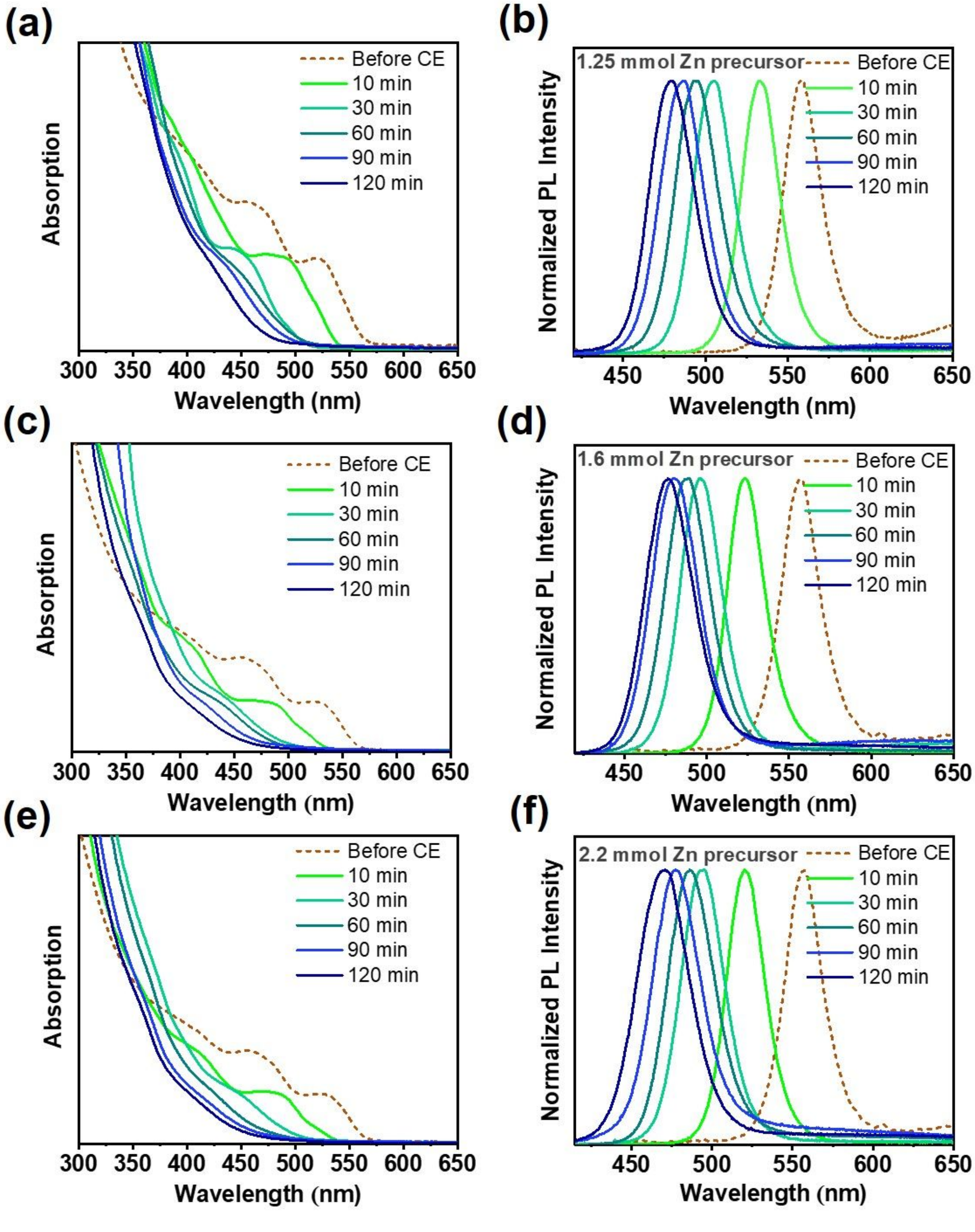

Figure S5. Evolution of the absorption and photoluminescence spectra of the 4 ML CdZnSeS/ZnS quaternary alloyed core/thin-shell NPLs during the 120 min CE reaction with different concentrations of ZnI_2: (a, b) low, (c, d) medium, and (e, f) high.

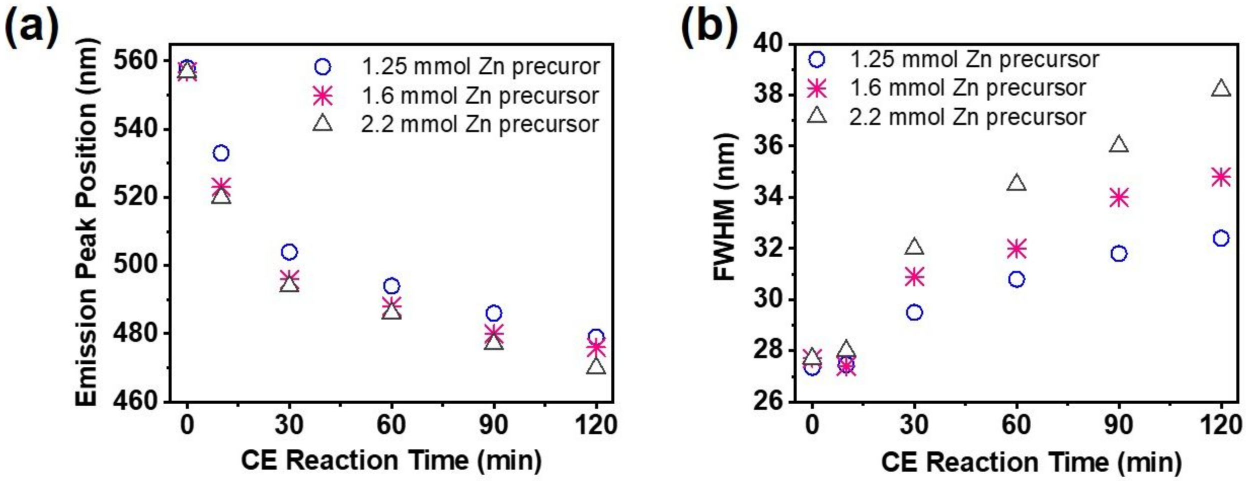

Figure S6. Variation in the (a) emission peak wavelength, and (b) FWHM values of the alloyed core/thin-shell NPLs during the 120 min Cd-to-Zn CE reaction with three different concentrations of ZnI_2.

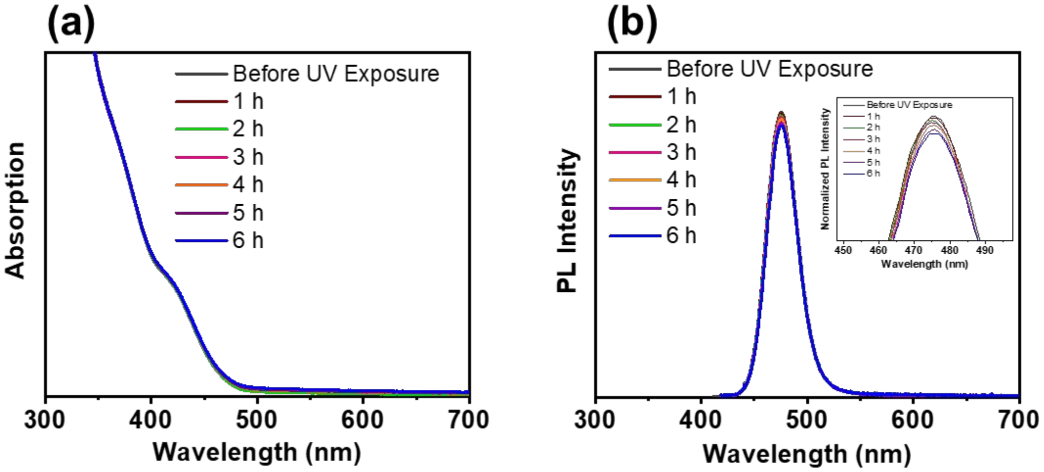

Figure S7. Evolution of (a) absorption spectra, and (b) PL spectra of the 4 ML CdZnSeS/ZnS quaternary alloyed core/thick-shell NPLs during continuous exposure to a 365 nm UV lamp at room temperature.

Table S1. Optical properties of the alloyed core, alloyed core/thin-shell, quaternary alloyed core/thin-shell, and quaternary core/thick-shell NPLs for the blue- and green-emitting series.

**Sample**	**PL emission wavelength (nm)**	**FWHM (nm)**	**PLQY (%)**
**$CdSe_{0.7}S_{0.3}$ Alloyed core NPLs**	486	15.0	25
**$CdSe_{0.7}S_{0.3}$/ZnS Alloyed core/thin-shell NPLs**	558	27.3	< 6
**Blue-emitting CdZnSeS/ZnS Quaternary alloyed core/thin-shell NPLs**	473	33.0	13
**Blue-emitting CdZnSeS/ZnS Quaternary alloyed core/thick-shell NPLs**	475	33.8	74
**Green-emitting CdZnSeS/ZnS Quaternary alloyed core/thin-shell NPLs**	501	29.7	10
**Green-emitting CdZnSeS/ZnS Quaternary alloyed core/thick-shell NPLs**	509	32.2	72

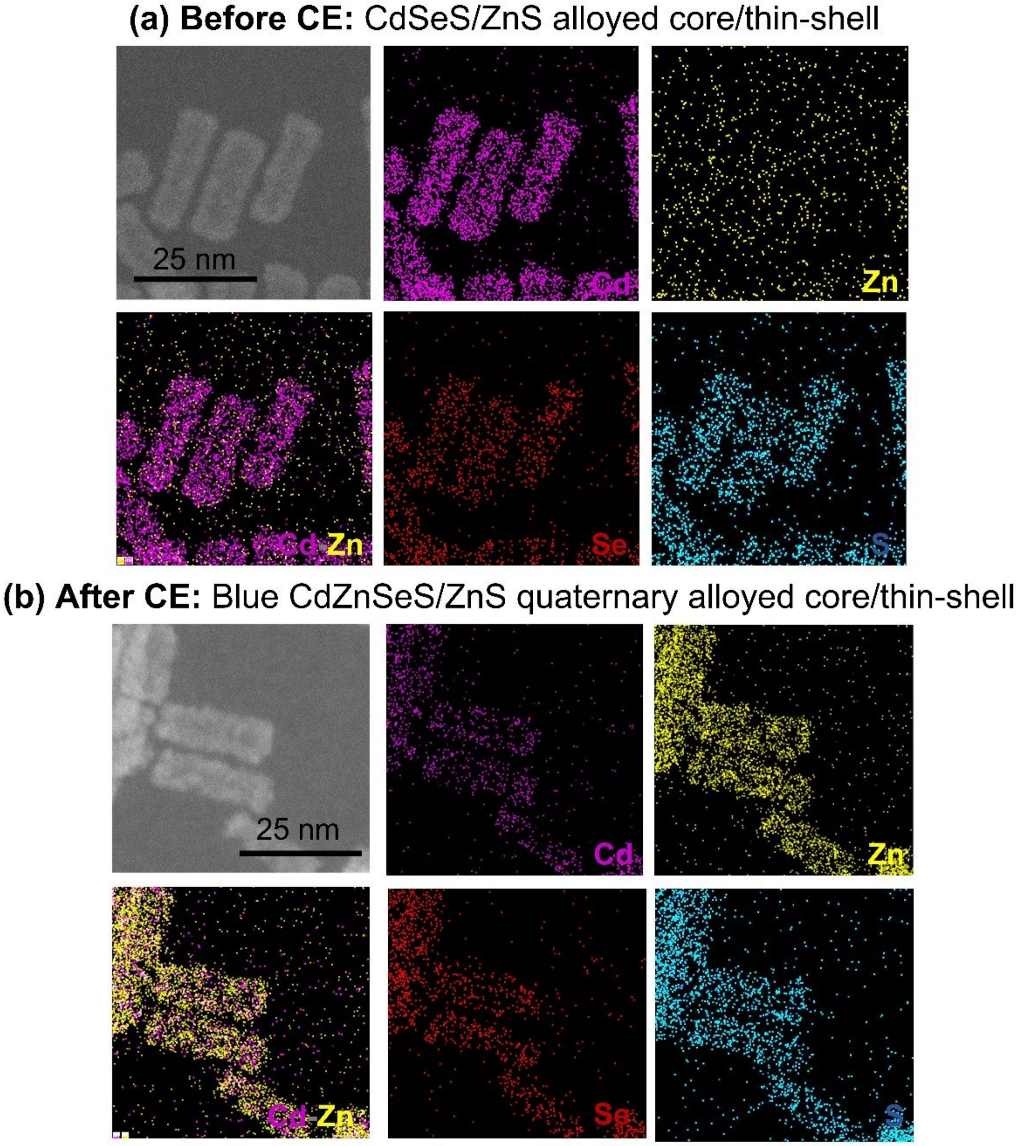

Figure S8. TEM-EDS mapping images of Cd, Zn, Se, and S for the blue-emitting NPLs before and after CE: (a) $CdSe_{0.7}S_{0.3}/ZnS$ alloyed core/thin-shell NPLs, and (b) CdZnSeS/ZnS quaternary alloyed core/thin-shell NPLs synthesized via 150 min CE at 310 ºC.

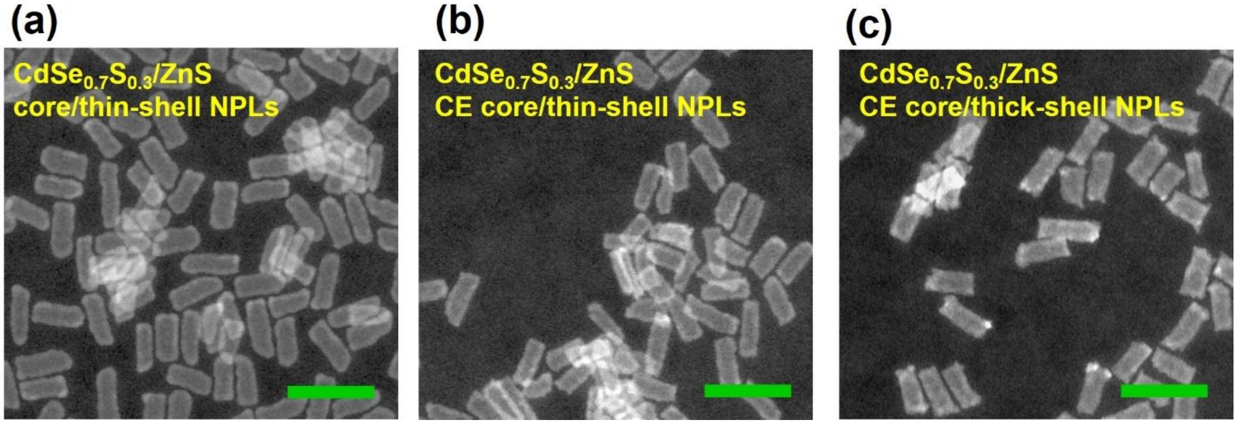

Figure S9. HAADF-STEM images of the green-emitting NPLs before and after CE reaction: (a) $CdSe_{0.7}S_{0.3}/ZnS$ alloyed core/thin-shell NPLs, (b) CdZnSeS/ZnS quaternary alloyed core/thin-shell NPLs synthesized via 30 min CE at 310 ºC, and (c) green-emitting CdZnSeS/ZnS quaternary alloyed core/thick-shell NPLs. Scale bars are 50 nm.

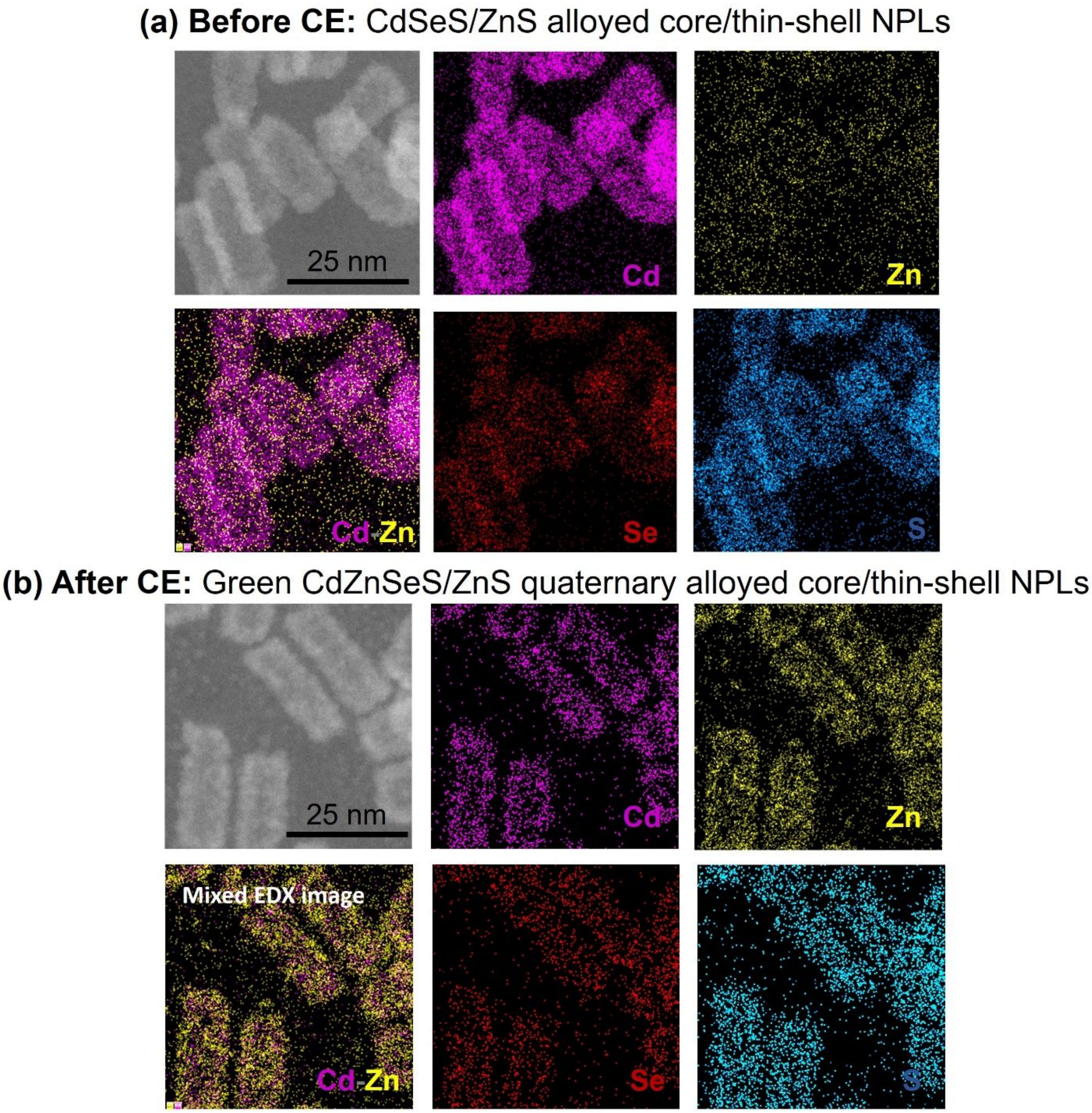

Figure S10. TEM-EDS mapping images of Cd, Zn, Se, and S for the green-emitting NPLs before and after CE: (a) $CdSe_{0.7}S_{0.3}/ZnS$ alloyed core/thin-shell NPLs, and (b) CdZnSeS/ZnS quaternary alloyed core/thin-shell NPLs synthesized via the 30 min CE at 310 ºC.

Table S2. XRD data of the alloyed core, alloyed core/thin-shell, quaternary alloyed core/thin-shell, and final thick-shelled NPLs for the blue-emitting series (150 min CE).

Sample	Plane	2Θ (degree)	d space (Å)	FWHM* (degree)
**4 ML $CdSe_{0.7}S_{0.3}$ Alloyed Core NPLs**	111	25.28	3.52	3.38
	200	29.45	3.03	
	220	41.92	2.15	
	311	49.88	1.83	
**$CdSe_{0.7}S_{0.3}/ZnS$ Alloyed core/thin-shell NPLs**	111	25.73	3.46	2.43
	200	30.09	2.97	
	220	42.99	2.10	
	311	50.78	1.80	
**Blue-emitting CdZnSeS/ZnS Quaternary alloyed core/thin-shell NPLs**	111	27.24	3.27	2.12
	200	32.09	2.79	
	220	45.98	1.97	
	311	54.38	1.69	
**Blue-emitting CdZnSeS/ZnS Quaternary alloyed core/thick-shell NPLs**	111	27.87	3.20	1.60
	200	32.44	2.76	
	220	46.54	1.95	
	311	55.16	1.66	

* Determined only for the (111) plane.

Table S3. XRD data of the alloyed core, alloyed core/thin-shell, quaternary alloyed core/thin-

**Sample**	**Plane**	**2Θ (degree)**	**d space (Å)**	**FWHM* (degree)**
**4 ML $CdSe_{0.7}S_{0.3}$ Alloyed Core NPLs**	111	25.16	3.54	2.99
	200	29.48	3.03	
	220	41.93	2.15	
	311	49.88	1.83	
**$CdSe_{0.7}S_{0.3}$/ZnS Alloyed core/thin-shell NPLs**	111	25.73	3.46	2.43
	200	30.09	2.97	
	220	42.99	2.10	
	311	50.78	1.80	
**Green-emitting CdZnSeS/ZnS Quaternary alloyed core/thin-shell NPLs**	111	26.86	3.32	2.24
	200	31.84	2.81	
	220	44.94	2.02	
	311	53.67	1.71	
**Green-emitting CdZnSeS/ZnS Quaternary alloyed core/thick-shell NPLs**	111	27.68	3.22	1.60
	200	32.09	2.79	
	220	46.43	1.95	
	311	55.32	1.66	

* Determined for the (111) diffraction peak.

shell, and final thick-shelled NPLs for the green-emitting series (30 min CE).

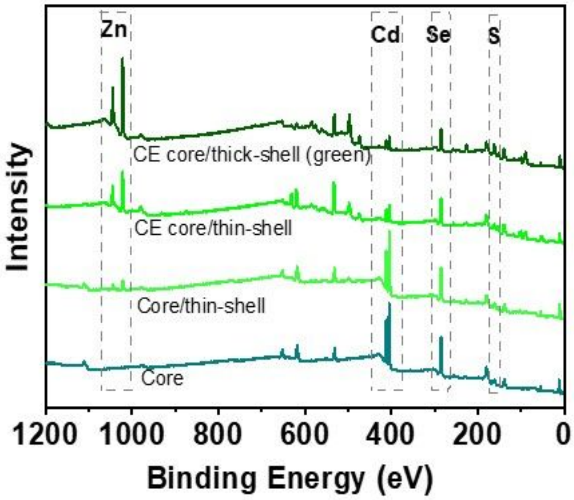

Figure S11. XPS survey spectra of the $CdSe_{0.7}S_{0.3}$ alloyed core, $CdSe_{0.7}S_{0.3}$/ZnS alloyed core/thin-shell, CdZnSeS/ZnS quaternary alloyed core/thin-shell, and the final CdZnSeS/ZnS quaternary alloyed core/thick-shell for the green-emitting NPLs (30 min CE).

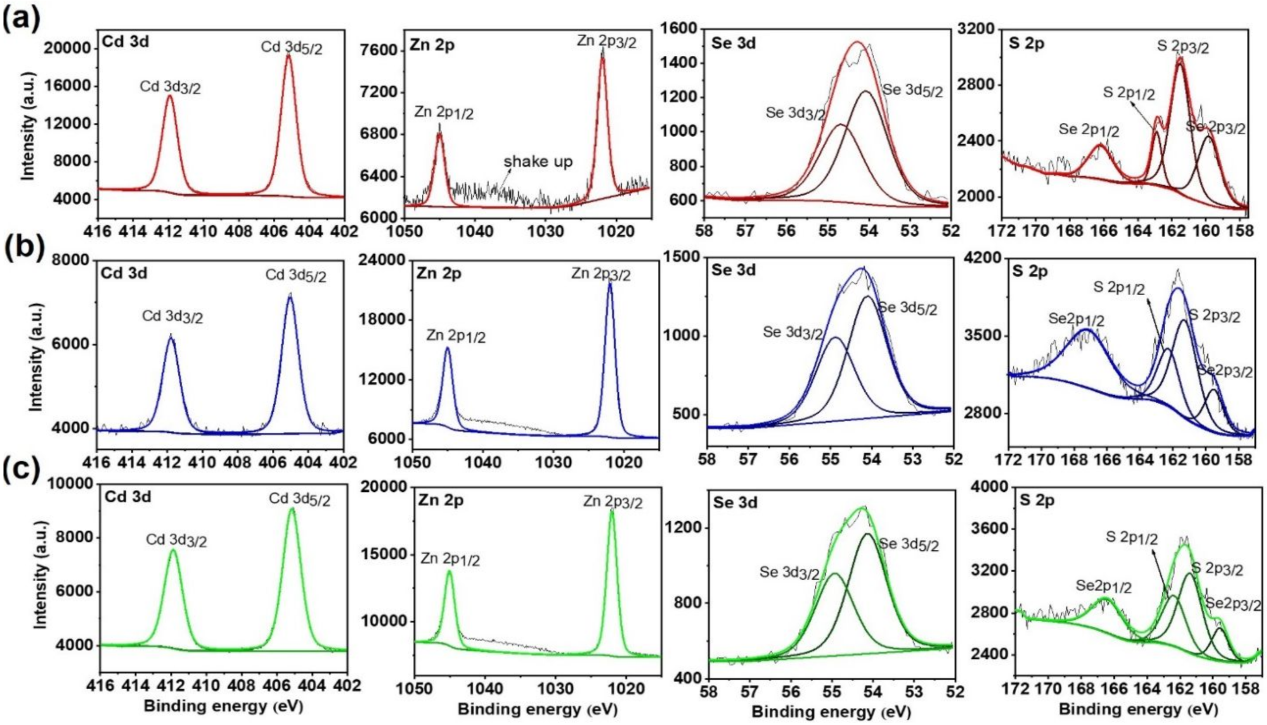

Figure S12. High-resolution XPS spectra of Cd 3d, Zn 2p, Se 3d, and S 2p for the synthesized NPLs before and after the Cd-to-Zn CE: (a) $CdSe_{0.7}S_{0.3}$/ZnS alloyed core/thin-shell, (b) blue-

emitting CdZnSeS/ZnS quaternary alloyed core/thin-shell synthesized via 150 min CE, and (c) green-emitting CdZnSeS/ZnS quaternary alloyed core/thin-shell synthesized via 30 min CE.

Table S4. XPS elemental composition of the synthesized NPLs before and after the Cd-to-Zn CE.

Sample	XPS Atomic percentage (%)			
	**Cd**	**Zn**	**Se**	**S**
**$CdSe_{0.7}S_{0.3}$/ZnS Alloyed core/thin-shell NPLs**	30.17	15.63	20.95	33.25
**Blue-emitting CdZnSeS/ZnS Quaternary alloyed core/thin-shell NPLs (150 min CE)**	4.04	52.38	16.02	27.56
**Blue-emitting CdZnSeS/ZnS Quaternary alloyed core/thick-shell NPLs**	2.01	54.27	8.72	35.00
**Green-emitting CdZnSeS/ZnS Quaternary alloyed core/thin-shell NPLs (30 min CE)**	6.99	53.13	13.74	26.13
**Green-emitting CdZnSeS/ZnS Quaternary alloyed core/thick-shell NPLs**	3.40	51.18	10.48	34.94

The number of ZnS shell layers in the core/thin-shell and core/thick-shell NPLs was determined by considering the XPS elementary compositions of the alloyed core/thin-shell, quaternary alloyed core/thin-shell (after CE), and the final thick-shelled NPLs, assuming that each core or shell layer has the same volume and contains an equal amount of cations, originated from their 2D planar geometry. The number of ZnS shell layers in the final blue-emitting quaternary core/thick-shell NPLs was evaluated to be ~3-4, with 1-2 layers deposited during the thin-shell growth step on the $CdSe_{0.7}S_{0.3}$ core NPLs.

Table S5. XPS elemental composition of CdSeS/ZnS alloyed core/thin-shell NPLs before CE and during the Cd-to-Zn CE reaction at intervals from 10 min to 210 min along with the corresponding Zn and Cd ratios calculated from these results.

**CdSeS/ZnS Alloyed core/thin shell NPLs**				
**Before CE**	Cd_{core}	Zn_{total}*	Cd content in NPL core	Zn content in NPL core
**---**	30.17	15.63	1	0
**Blue-emitting CdZnSeS/ZnS Quaternary alloyed core/thin shell NPLs**				
**CE reaction time (min)**	Cd_{core}	Zn_{total}*	Cd content in NPL core	Zn content in NPL core
**10**	9.48	45.50	0.300	0.700
**30**	6.99	53.13	0.204	0.796
**60**	5.47	52.87	0.164	0.836
**90**	5.01	53.83	0.149	0.851
**120**	4.41	54.35	0.131	0.869
**150**	4.02	55.35	0.120	0.880
**210**	3.18	54.91	0.096	0.904

* $Zn_{total}=Zn_{core}+Zn_{shell}$

Using the XPS elementary composition of Cd and Zn after different CE reaction times, we calculated the concentration of each cation in the core of the cation-exchanged NPLs by knowing the concentration of each cations in each sample and considering the fact that each core or shell layer has the same volume and same amount of cations originated from their 2D planar geometry. The NPLs were washed two times with hexane/ethanol before XPS measurements.

Table S6. TRF decay components of the alloyed core, core/thin-shell, quaternary alloyed core/thin-shell, and final thick-shelled NPLs for the blue-emitting series, (150 min CE).

**4ML $CdSe_{0.7}S_{0.3}$ Alloyed core NPLs**						
τ_1 (ns)	τ_2 (ns)	τ_3 (ns)	$A_1.\tau_1$ (%)	$A_2.\tau_2$ (%)	$A_3.\tau_3$ (%)	$\tau_{int.}$* (ns)
0.8	10.2	85.0	0.2	0.4	0.3	**34.2**
**$CdSe_{0.7}S_{0.3}$/ZnS Alloyed core/thin-shell NPLs**						
τ_1 (ns)	τ_2 (ns)	τ_3 (ns)	$A_1.\tau_1$ (%)	$A_2.\tau_2$ (%)	$A_3.\tau_3$ (%)	$\tau_{int.}$* (ns)
0.4	6.5	85.7	0.1	0.2	0.7	**62.7**

Blue-emitting CdZnSeS/ZnS Quaternary alloyed core/thin-shell NPLs						
τ_1 (ns)	τ_2 (ns)	τ_3 (ns)	$A_1.\tau_1$ (%)	$A_2.\tau_2$ (%)	$A_3.\tau_3$ (%)	$\tau_{int.}$* (ns)
0.9	9.0	81.9	0.1	0.4	0.5	**46.9**
**Blue-emitting CdZnSeS/ZnS Quaternary alloyed core/thick-shell NPLs**						
τ_1 (ns)	τ_2 (ns)	τ_3 (ns)	$A_1.\tau_1$ (%)	$A_2.\tau_2$ (%)	$A_3.\tau_3$ (%)	$\tau_{int.}$* (ns)
9.1	43.8	280.5	0.4	0.4	0.2	**77.8**

* $\tau_{int.}$: Intensity-averaged lifetime

Intensity-averaged lifetime is calculated by:[1]

$$\tau_{int.} = \frac{\sum A_i \tau_i^2}{\sum A_i \tau_i}, \qquad \text{(Eq. S1)}$$

Where A_i is the amplitude (pre-exponential factor) of the i-th component, and τ_i is the lifetime of the i-th component

Table S7. TRF decay components of the alloyed core, core/thin-shell, CE core/thin-shell and the final thick-shelled NPLs for the green-emitting series (30 min CE at 310 °C).

4ML $CdSe_{0.7}S_{0.3}$ Alloyed core NPLs						
τ_1 (ns)	τ_2 (ns)	τ_3 (ns)	$A_1.\tau_1$ (%)	$A_2.\tau_2$ (%)	$A_3.\tau_3$ (%)	$\tau_{int.}$* (ns)
1.3	8.3	50.0	0.2	0.4	0.5	**26.7**
**$CdSe_{0.7}S_{0.3}$/ZnS Alloyed core/thin-shell NPLs**						
τ_1 (ns)	τ_2 (ns)	τ_3 (ns)	$A_1.\tau_1$ (%)	$A_2.\tau_2$ (%)	$A_3.\tau_3$ (%)	$\tau_{int.}$* (ns)

1.0	9.9	82.5	0.1	0.3	0.6	**53.8**
**Green-emitting CdZnSeS/ZnS Quaternary alloyed core/thin-shell NPLs**						
τ_1 (ns)	τ_2 (ns)	τ_3 (ns)	$A_1.\tau_1$ (%)	$A_2.\tau_2$ (%)	$A_3.\tau_3$ (%)	$\tau_{int.}$* (ns)
0.7	7.9	79.2	0.1	0.4	0.5	**45.2**
**Green-emitting CdZnSeS/ZnS Quaternary alloyed core/thick-shell NPLs**						
τ_1 (ns)	τ_2 (ns)	τ_3 (ns)	$A_1.\tau_1$ (%)	$A_2.\tau_2$ (%)	$A_3.\tau_3$ (%)	$\tau_{int.}$* (ns)
5.9	20.7	171.9	0.1	0.5	0.3	**70.5**

* $\tau_{int.}$: Intensity-averaged lifetime

Intensity-averaged lifetime ($\tau_{int.}$) is calculated by Eq. S1.

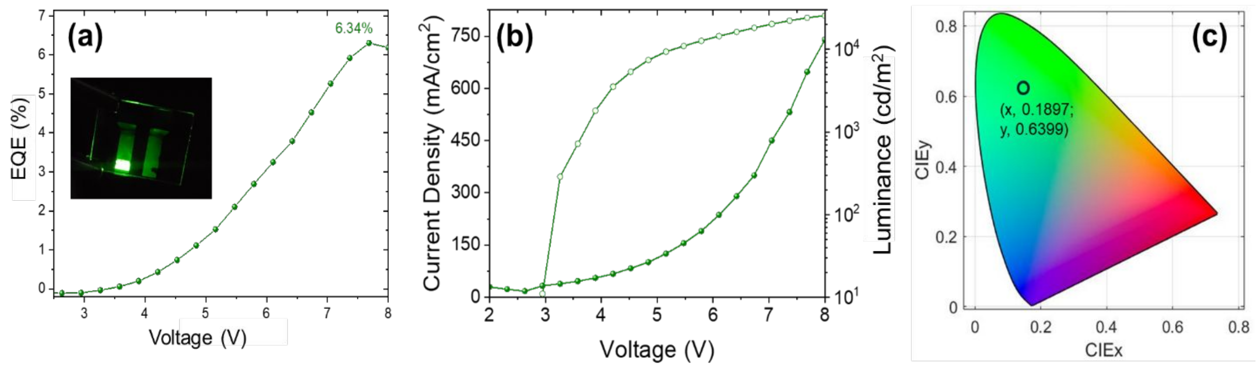

Figure S13. (a) EQE versus driving voltage (the inset image shows the fabricated NPL-LEDs at 7 V), (b) Current density-luminance-voltage characteristics, and (c) CIE gamut. All for the green-

emitting NPL-LED with green emitting CdZnSeS/ZnS quaternary alloyed core/thick-shell NPLs as the active material.

Table S8. Comparison of device properties of our developed blue and green NPL-LEDs and other reported blue and green LEDs

Emitter	EL λ_{max} (nm)	PL λ_{max} (nm)	PLQY (%)	L_{max} (Cd m^{-2})	EQE_{max} (%)	V_T (V)	Year[Ref.]
**Blue-emitting Perovskites-LEDs**							
$CsPbBr_3$ NPLs	469	465	69	41.8	1.42	3	2019[2]
$ZFAPbBr_3$ NPLs	439	55	NA	NA	0.14	3.6	2020[3]
$CsPbBr_3$ NPLs	465	457	40	631	0.8	NA	2021[4]
$CsPbBr_3$ NPLs	462	460	NA	691	1.77	NA	2023[5]
$CsPbBr_3$/$FAPbBr_3$ core/crown NPLs	457	NA	25	513	0.4	4	2023[6]
$CsPbBr_xCl_{3-x}$	472	468	87	275	3.2	4	2024[7]
**Blue-emitting QDs-LEDs**							
CdS/ZnS NC QDs	460	NA	NA	1600	5	5.5	2007[8]
ZnCdS/ZnS core/shell QDs	443	441	NA	2600	10.3	2.6	2015[9]
InP/GaP/ZnS QDs	488	480	81	3120	1	NA	2020[10]
ZnSe/ZnS	446	443	92	710	2.25	5.1	2024[11]
**Blue-emitting NPLs-LEDs**							
Cyan-emitting $CdSe_{1-x}S_x$ core NPL	496	491	NA	100	NA	2.1	2015[12]
Cyan-emitting 4.5 ML CdZnSe/ZnS core/shell NPLs	498	492	<60	11,400	NA	2.25	2022[13]
4 ML CdSeS/CdS core/crown NPLs	476	468	60	12	0.06	4	2022[1]
3.5 ML CdSe/CdS core/crown NPLs	461	450	90	46	1.16	3.6	2022[14]
CdSe/CdS core/crown NPLs	467	465	66	150	1.3	2.9	2025[15]
CdZnSe/ZnS core/shell NPLs	476	NA	~60	38,063	10.4	3.5	2025[16]
**Green-emitting NPLs-LEDs**							
4 ML CdSe/CdS core/crown NPLs	516	512	47	1,036	0.32	2.96	2019[17]
4 ML CdSeS/ZnS core/shell NPLs	558	554	NA	21,280	2.4	2.4	2020[18]
CdSe/CdS core/crown NPLs	522	517	80	22,400	2.16	2.1	2021[19]
4.5 ML CdZnSe/ZnS core/shell NPLs	544	538	66	28,200	NA	2.3	2022[13]
CdSe/CdSeS core/alloyed-crown	532	528	NA	600	9.78	3	2022[20]
CdZnSe/ZnS core/shell NPLs	527	NA	~80	347,683	20.6	3	2025[16]
CdZnSeS/ZnS Quaternary alloyed core/thick-shell NPLs	482	475	74	12,451	11.3	<2.5	This work
	516	509	72	13,990	6.3	2.7	

References

(1) İzmir, M.; Sharma, A.; Shendre, S.; Durmusoglu, E. G.; Sharma, V. K.; Shabani, F.; Baruj, H. D.; Delikanli, S.; Sharma, M.; Demir, H. V. Blue-Emitting CdSe Nanoplatelets Enabled by Sulfur-Alloyed Heterostructures for Light-Emitting Diodes with Low Turn-on Voltage. *ACS Appl. Nano Mater.* **2021,** *5*, 1367-1376.

(2) Zhang, C.; Wan, Q.; Wang, B.; Zheng, W.; Liu, M.; Zhang, Q.; Kong, L.; Li, L. Surface Ligand Engineering toward Brightly Luminescent and Stable Cesium Lead Halide Perovskite Nanoplatelets for Efficient Blue-Light-Emitting Diodes. *J. Phys. Chem. C* **2019,** *123*, 26161-26169.

(3) Peng, S.; Wen, Z.; Ye, T.; Xiao, X.; Wang, K.; Xia, J.; Sun, J.; Zhang, T.; Mei, G.; Liu, H. Effective Surface Ligand-Concentration Tuning of Deep-Blue Luminescent $FAPbBr_3$ Nanoplatelets with Enhanced Stability and Charge Transport. *ACS Appl. Mater. Interfaces* **2020,** *12*, 31863-31874.

(4) Yin, W.; Li, M.; Dong, W.; Luo, Z.; Li, Y.; Qian, J.; Zhang, J.; Zhang, W.; Zhang, Y.; Kershaw, S. V. Multidentate Ligand Polyethylenimine Enables Bright Color-Saturated Blue Light-Emitting Diodes Based on $CsPbBr_3$ Nanoplatelets. *ACS Energy Lett.* **2021,** *6*, 477-484.

(5) Liu, H.; Worku, M.; Mondal, A.; Shonde, T. B.; Chaaban, M.; Ben-Akacha, A.; Lee, S.; Gonzalez, F.; Olasupo, O.; Lin, X. Efficient and Stable Blue Light Emitting Diodes Based on $CsPbBr_3$ Nanoplatelets with Surface Passivation by a Multifunctional Organic Sulfate. *Adv. Energy Mater.* **2023,** *13*, 2201605.

(6) Kshirsagar, A. S.; Gangishetty, M. K. In Situ Controlled Growth of Strongly Quantum Confined $CsPbBr_3$/$FAPbBr_3$ Core/Crown Nanoplatelets for Blue Light Emitting Diodes. *Adv. Opt. Mater.* **2023,** *11*, 2301343.

(7) Chen, T.; Ru, X.-C.; Ma, Z.-Y.; Feng, L.-Z.; Song, K.-H.; Ge, J.; Zhu, B.-S.; Yang, J.-N.; Yao, H.-B. Tetrafluoroborate-Passivated $CsPbBr_xCl_{3-x}$ Nanocrystals for Spectrally Stable Pure Blue Perovskite Light-Emitting Diodes. *ACS Appl. Nano Mater.* **2024,** *7*, 4474-4480.

(8) Tan, Z.; Zhang, F.; Zhu, T.; Xu, J.; Wang, A. Y.; Dixon, J. D.; Li, L.; Zhang, Q.; Mohney, S. E.; Ruzyllo, J. Bright and Color-Saturated Emission from Blue Light-Emitting Diodes Based on Solution-Processed Colloidal Nanocrystal Quantum Dots. *Nano Lett.* **2007,** *7*, 3803-3807.

(9) Shen, H.; Cao, W.; Shewmon, N. T.; Yang, C.; Li, L. S.; Xue, J. High-Efficiency, Low Turn-on Voltage Blue-Violet Quantum-Dot-Based Light-Emitting Diodes. *Nano Lett.* **2015,** *15*, 1211-1216.

(10) Zhang, H.; Ma, X.; Lin, Q.; Zeng, Z.; Wang, H.; Li, L. S.; Shen, H.; Jia, Y.; Du, Z. High-Brightness Blue InP Quantum Dot-Based Electroluminescent Devices: The Role of Shell Thickness. *J. Phys. Chem. Lett.* **2020,** *11*, 960-967.

(11) Zheng, Z.; Ren, Z.; Liu, Y.; Yuan, Y.; Zhou, X.; Ji, H.; Shi, H.; Zhang, Y.; Chen, Y.; Zhao, Q. Blue-Emitting ZnSeTe/ZnSe/ZnS Quantum Dots for Efficient Electroluminescent Application. *ACS Appl. Nano Mater.* **2024,** *7*, 13166-13172.

(12) Fan, F.; Kanjanaboos, P.; Saravanapavanantham, M.; Beauregard, E.; Ingram, G.; Yassitepe, E.; Adachi, M. M.; Voznyy, O.; Johnston, A. K.; Walters, G. Colloidal $CdSe_{1-x}S_x$

Nanoplatelets with Narrow and Continuously-Tunable Electroluminescence. *Nano Lett.* **2015,** *15*, 4611-4615.

(13) Yoon, D.-E.; Yeo, S.; Lee, H.; Cho, H.; Wang, N.; Kim, G.-M.; Bae, W. K.; Lee, Y. K.; Park, Y.-S.; Lee, D. C. Pushing the Emission Envelope for Full-Color Realization of Colloidal Semiconductor Core/Shell Nanoplatelets. *Chem. Mater.* **2022,** *34*, 9190-9199.

(14) Hu, A.; Bai, P.; Zhu, Y.; Tang, Z.; Xiao, L.; Gao, Y. Controlled Core/Crown Growth Enables Blue-Emitting Colloidal Nanoplatelets with Efficient and Pure Photoluminescence. *Small* **2022,** *18*, 2204120.

(15) Cirignano, M.; Roshan, H.; Farinini, E.; Di Giacomo, A.; Fiorito, S.; Piccinotti, D.; Khabbazabkenar, S.; Di Stasio, F.; Moreels, I. Blue CdSe/CdS Core/Crown Nanoplatelet Light-Emitting Diodes Obtained via a Design-of-Experiments Approach. *Nanoscale* **2025,** *17*, 304-313.

(16) Zhu, Y.; Lu, X.; Qiu, J.; Bai, P.; Hu, A.; Yao, Y.; Liu, Q.; Li, Y.; Yu, W.; Li, Y. High-Performance Green and Blue Light-Emitting Diodes Enabled by CdZnSe/ZnS Core/Shell Colloidal Quantum Wells. *Adv. Mater.* **2025,** *37*, 2414631.

(17) Yang, Y.; Zhang, C.; Qu, X.; Zhang, W.; Marus, M.; Xu, B.; Wang, K.; Sun, X. W. High Quantum Yield Colloidal Semiconducting Nanoplatelets and High Color Purity Nanoplatelet QLED. *IEEE T. Nanotechnol.* **2019,** *18*, 220-225.

(18) Altintas, Y.; Liu, B.; Hernandez-Martinez, P. L.; Gheshlaghi, N.; Shabani, F.; Sharma, M.; Wang, L.; Sun, H.; Mutlugun, E.; Demir, H. V. Spectrally Wide-Range-Tunable, Efficient, and Bright Colloidal Light-Emitting Diodes of Quasi-2D Nanoplatelets Enabled by Engineered Alloyed Heterostructures. *Chem. Mater.* **2020,** *32*, 7874-7883.

(19) Wen, Z.; Liu, P.; Ma, J.; Jia, S.; Xiao, X.; Ding, S.; Tang, H.; Yang, H.; Zhang, C.; Qu, X. High-Performance Ultrapure Green CdSe/CdS Core/Crown Nanoplatelet Light-Emitting Diodes by Suppressing Nonradiative Energy Transfer. *Adv. Electron. Mater.* **2021,** *7*, 2000965.

(20) Bai, P.; Hu, A.; Deng, Y.; Tang, Z.; Yu, W.; Hao, Y.; Yang, S.; Zhu, Y.; Xiao, L.; Jin, Y. CdSe/CdSeS Nanoplatelet Light-Emitting Diodes with Ultrapure Green Color and High External Quantum Efficiency. *J. Phys. Chem. Lett.* **2022,** *13*, 9051-9057.
